# Functional Variants in *DPYSL2* Sequence Increase Risk of Schizophrenia and Suggest a Link to mTOR Signaling

**DOI:** 10.1534/g3.114.015636

**Published:** 2014-11-20

**Authors:** Yaping Liu, Xuan Pham, Lilei Zhang, Pei-lung Chen, Grzegorz Burzynski, David M. McGaughey, Shan He, John A. McGrath, Paula Wolyniec, Margaret D. Fallin, Megan S. Pierce, Andrew S. McCallion, Ann E. Pulver, Dimitrios Avramopoulos, David Valle

**Affiliations:** * McKusick-Nathans Institute of Genetic Medicine, Johns Hopkins University School of Medicine and, Johns Hopkins Bloomberg School of Public Health, Baltimore, Maryland 21205; † Predoctoral Training Program in Human Genetics, Johns Hopkins University School of Medicine and, Johns Hopkins Bloomberg School of Public Health, Baltimore, Maryland 21205; ‡ Department of Molecular Biology and Genetics, Johns Hopkins University School of Medicine and, Johns Hopkins Bloomberg School of Public Health, Baltimore, Maryland 21205; § Department of Psychiatry, Johns Hopkins University School of Medicine and, Johns Hopkins Bloomberg School of Public Health, Baltimore, Maryland 21205; ** Department of Epidemiology, Johns Hopkins University School of Medicine and, Johns Hopkins Bloomberg School of Public Health, Baltimore, Maryland 21205; †† Department of Molecular and Comparative Pathobiology, Johns Hopkins University School of Medicine and, Johns Hopkins Bloomberg School of Public Health, Baltimore, Maryland 21205

**Keywords:** gene regulation, brain development, RNA translation, CRMP2, mTOR

## Abstract

Numerous linkage and association studies by our group and others have implicated *DPYSL2* at 8p21.2 in schizophrenia. Here we explore *DPYSL2* for functional variation that underlies these associations. We sequenced all 14 exons of *DPYSL2* as well as 27 conserved noncoding regions at the locus in 137 cases and 151 controls. We identified 120 variants, eight of which we genotyped in an additional 729 cases and 1542 controls. Several were significantly associated with schizophrenia, including a three single-nucleotide polymorphism (SNP) haplotype in the proximal promoter, two SNPs in intron 1, and a polymorphic dinucleotide repeat in the 5′-untranslated region that alters sequences predicted to be involved in translational regulation by mammalian target of rapamycin signaling. The 3-SNP promoter haplotype and the sequence surrounding one of the intron 1 SNPs direct tissue-specific expression in the nervous systems of Zebrafish in a pattern consistent with the two endogenous *dpysl2* paralogs. In addition, two SNP haplotypes over the coding exons and 3′ end of *DPYSL2* showed association with opposing sex-specific risks. These data suggest that these polymorphic, schizophrenia-associated sequences function as regulatory elements for *DPYSL2* expression. In transient transfection assays, the high risk allele of the polymorphic dinucleotide repeat diminished reporter expression by 3- to 4-fold. Both the high- and low-risk alleles respond to allosteric mTOR inhibition by rapamycin until, at high drug levels, allelic differences are eliminated. Our results suggest that reduced transcription and mTOR-regulated translation of certain *DPYSL2* isoforms increase the risk for schizophrenia.

Schizophrenia (SZ; MIM#181500) is a disabling neuropsychiatric disorder with onset in young adult life and a worldwide incidence of 1% (Eaton 1985). Current models suggest that SZ is a neurodevelopmental disorder with a strong genetic component (Sawa and Snyder 2002; Aleman *et al.* 2003; Insel 2010). Interestingly, there are multiple unexplained sex-specific phenotypic differences in SZ. Females are less frequently affected and comprise only ~30% of the cases in most series (Hafner *et al.* 1993; McGrath *et al.* 2004). The average age of onset in females is late 20s compared with early 20s in males (Goldstein *et al.* 1990; Hafner 2003). SZ in females also demonstrates a greater degree of familiality (Shtasel *et al.* 1992) and more affective symptoms (Szymanski *et al.* 1995; Sawa and Snyder 2002).

Efforts to identify SZ susceptibility loci (SSL) have been complicated by multiple factors, including phenotypic variation (Jablensky 2006), diagnostic imprecision (Cardno *et al.* 2002), and locus and allelic heterogeneity (Owen *et al.* 2007; Insel 2010). Despite these challenges, positional and functional studies have revealed multiple genes with alleles that contribute risk for SZ including, *DTNBP1* in 6p22 (Straub *et al.* 2002), *DAOA* in 13q33 (Chumakov *et al.* 2002), *DISC1* in 1q42 1 (St Clair *et al.* 1990; Millar *et al.* 2000), *NRG1* in 8p21, (Stefansson *et al.* 2002) and *NRG3* in 10q22 (Chen *et al.* 2009; Kao *et al.* 2010; Morar *et al.* 2011). Recent hypothesis-free genome-wide association studies and studies of *de novo* events are adding to this list of genes [Schizophrenia Psychiatric Genome-Wide Association Study (GWAS) Consortium 2011; Bergen and Petryshen 2012; Rees *et al.* 2012; Xu *et al.* 2012; Schizophrenia Working Group of the Psychiatric Genomics Consortium 2014]. The evidence for SSL on chromosome 8p originated with a series of linkage studies from our group (Pulver *et al.* 1995, 2000; Blouin *et al.* 1998;) that contributed to the identification of *NRG1* located at 8p12 (32.5–32.8 Mb) as an SSL in the Icelandic population (Stefansson *et al.* 2002). Subsequently, this result has been replicated in many other populations (Li *et al.* 2006). However, subsequent linkage and a linkage meta-analysis studies found the strongest evidence for linkage on 8p telomeric to *NRG1* (Holmans *et al.* 2009; Ng *et al.* 2009). Holmans *et al.* (2009) performed the largest SZ linkage study to date with 807 families, including 124 European Caucasian (CEU) families from our group. The peak linkage signal from the entire sample was a parametric heterogeneity LOD score of 2.76 at rs1561817 (chr8: 26,591,503; hg18), located ~4 Mb telomeric of *NRG1 and only* ~20 kb centromeric of *DPYSL2*. The peak linkage signal from our families alone was at rs388047 in intron 2 of *DPYSL2*. These results agree with our earlier candidate gene-based association study in an Ashkenazi Jewish (AJ) SZ cohort that also implicated *DPYSL2* as a risk gene for SZ (Fallin *et al.* 2005). Prompted by this evidence converging on the region in and around *DPYSL2*, we subsequently performed a single-nucleotide polymorphism (SNP) fine mapping association study with 1064 SNPs distributed over ~2.8 Mb of 8p, including *DPYSL2* in an AJ population. We found the most significant associations with SZ at SNPs rs12155555 (*P* = 6 × 10^−5^) and rs5029306 (*P* = 2.3 × 10^-4^), located ~5 kb 3′ and within intron 3, respectively of *DPYSL2* (Fallin *et al.* 2011). These SNPs are not in linkage disequilibrium (LD) with each other. In that study we also found possible sex effects. Here we follow up on that study, looking for the functional variants that might underlie the association of rs12155555 and rs5029306.

Expressed in both the central nervous system (CNS) and PNS, *DPYSL2* encodes the collapsin response mediator protein 2 or CRMP2, a 62-kDa cytosolic protein that plays a role in axon specification and growth (Arimura *et al.* 2004). There are three *DPYSL2* transcripts annotated in RefSeq, a shorter version (NM_001386, *DPYSL2B*), which was annotated first and has protein evidence (Owen *et al.* 2007; Insel 2010); a longer transcript (NM_001197293, *DPYSL2A*) with an alternative first exon located ~60 kb upstream of the 5′ end of *DPYSL2B* described by Johnston-Wilson *et al.* (2000); and a recently annotated transcript (NM_001244604, *DPYSL2C*) similar to the short transcript but initiated and translated further downstream from an in-frame start codon. This last transcript was not known at the time of this work and will not be discussed further. The *DPYSL2A* and *DPYSL2B* transcripts differ in their promoters, first exons, and N-terminal amino acid sequence of the CRMP2 proteins they encode. The annotated “short” *DPYSL2B* transcript encodes CRMP2B, a protein of 572 amino acids (AA) whereas the unannotated “long” *DPYSL2A* transcript encodes CRMP2A, a protein of 677 AA. The two CRMP2 isoforms have an identical C-terminal 559 AA sequence and differ only in their N-termini: CRMP2A has a predicted N-terminal sequence of 118 AA distinct from the 13 AA sequence of CRMP2B N-terminus. The long transcript *DPYSL2A* (AB209195.1) has not been validated at protein level. Little is known about *DPYSL2A* or CRMP2A but for *DPYSL2B*, multiple studies in cultured rat hippocampal neurons have shown that overexpression of the wild-type allele induces excessive arborization of axons while expression of a dominant negative allele inhibits the axonal outgrowth (Inagaki *et al.* 2001). CRMP2B function also has been implicated recently in the control of dendritic field projections (Yamashita *et al.* 2012). Multiple proteomic studies have found altered levels of CRMP2B in the brains of patients with SZ (Beardsley 1996; Edgar *et al.* 2000; Johnston-Wilson *et al.* 2000; Martins-de-Souza *et al.* 2009).

In the present study, we identify by targeted sequencing of the 14 *DPYSL2* exons and 27 nearby conserved noncoding regions (cNCRs) DNA variants that could underlie the associations of rs12155555 or rs5029306. We consider these variants likely to be functional and responsible for the observed associations, and we follow them up with *in vivo*, *in vitro*, and postmortem tissue assays to further support their possible functional relevance and importance in SZ. Our results identify multiple neuronal enhancers that carry sequence variation in and around *DPYSL2* and suggest a role for a polymorphic dinucleotide repeat (DNR) tract in the 5′-untranslated region (5′-UTR) of *DPYSL2B* in SZ and a link to mammalian target of rapamycin (mTOR) signaling.

## Materials and Methods

### Study design

In this study we follow up in our previous report of an association of rs12155555 and rs5029306 in the *DPYSL2* gene (Fallin *et al.* 2011), looking for the functional variants that underlie that association. We performed targeted sequencing for potentially functional variants that segregate with the risk haplotype. Variants were selected from those identified based on evidence of association with SZ due to their LD with rs12155555 and/or rs5029306 (note that for many variants reported here this evidence is expected to be weaker than the parent study of Fallin *et al.* 2011 which used a larger sample). We then performed functional testing of these variants *in vitro* (dual luciferase assays) and *in vivo* [zinc finger (ZF) transgenesis], and assessed their correlation with *DPYSL2* transcript levels in postmortem brain,

### Samples

Although the strongest association signals in our original study were in an AJ population, we also identified strong associations near *DPYSL2* in our Caucasian cohort (Fallin *et al.* 2011). For this reason, we decided to sequence samples from both populations.

#### European-Caucasian samples:

Our CEU family sample included 48 SZ probands and 96 CEPH controls (Supporting Information, Table S1). The families were recruited from multiple sources including our Maryland Epidemiologic Samples (Pulver and Bale 1989), our nationwide advertising efforts, and national and international collaborators (United States, Italy, Poland, Greece). All families are of European-Caucasian ancestry. Descriptions of our clinical assessment methods are published elsewhere (Blouin *et al.* 1998).

#### Ashkenazi samples:

We sequenced a total of 89 AJ SZ subjects from our previously described cohort that met probable or definite *Diagnostic and Statistical Manual of Mental Disorders*, Fourth Edition, SZ or schizoaffective disorder criteria based on a consensus diagnosis (Fallin *et al.* 2011). Subjects had a positive family history for psychosis, *i.e.*, at least two members of the family (the proband and one more first and second degree relatives) had evidence of psychosis either from direct assessment or from family informant data. We also sequenced 55 AJ controls from the same cohort, 48 of whom were older than 40 years of age (an age at which the risk of developing SZ is reduced by 90%). These 55 AJ controls had no history of depression, mania, psychosis, psychiatric hospitalization, depression, or suicide attempts. Of the 89 AJ SZ subjects, 41 were SZ probands who were homozygous for the risk alleles at both peak-associated SNPs (rs12155555; rs5029306). And of the 55 AJ controls, seven were homozygous for the nonrisk alleles at both rs12155555 and rs5029306 to identify variants segregating on the risk haplotype and to detect variants that might be specific to the 41 AJ SZ probands. Our AJ SZ and controls were recruited in North America by advertisements in newspapers and Jewish newsletters, talks to community organizations, letters to leaders of the Jewish community, letters and talks to service providers, and a study Web site hosted by the Johns Hopkins Epidemiology-Genetics Program (EpiGen) in Psychiatry (Fallin *et al.* 2003). AJ SZ cases and controls self-identified AJ ethnicity in all four grandparents. In addition to sequencing, we also conducted genotyping assays on 729 AJ SZ independent probands and 1542 AJ controls for 12 SNPs within and flanking *DPYSL2*. The 729 AJ SZ probands were all selected from the Epidemiology Genetics (Epigen) Program collection (Fallin *et al.* 2003); of the 1542 AJ control DNA samples, 821 were from Johns Hopkins EpiGen collection and 721 were from the New York Cancer Project Biorepository (Foulkes *et al.* 2002).

All recruitment methods and protocols for collection of clinical data and blood samples were approved by the Johns Hopkins institutional review board, and informed consent was obtained from all subjects.

### Sequencing

We amplified 14 exons of *DPYSL2* with flanking splice sites and 27 cNCRs in and around *DPYSL2* from 48 CEU and 89 AJ SZ subjects and from 55 AJ and 96 CEU controls. We defined the cNCRs as intergenic/intronic elements with PhastCons lod scores ranging from 13 to 927 (PhastCons Conserved Elements, 44-way Vertebrate Multiz Alignment in UCSC Genome Browser) (Siepel *et al.* 2005), and/or high 7X regulatory potential scores. The cNCRs were chosen to be in proximity to the peak SNPs shown in our previous linkage and association studies (Beasley *et al.* 2006). We Sanger sequenced all amplicons in both directions on an ABI 3100 DNA Sequencer and analyzed the results using CodonCode Aligner software (CodonCode Aligner Corporation). The polymerase chain reaction (PCR) primers are listed in Table S2. The 27 sequenced cNCRs were identified according to their position (kb) upstream (−) or downstream (+) to the *DPYSL2* (RefSeq: NM_001386) transcription start site or within *DPYSL2* introns (I), and are displayed as custom tracks on the UCSC genome browser (build 36) (Figure S1).

### SNP selection and genotyping

We selected14 SNPs for further study and confirmation of their association by genotyping in the extended AJ cohort. Of these, two had been previously genotyped in this sample (Fallin *et al.* 2011), rs12155555 and rs5029306. Another 10 SNPs were selected from those identified via sequencing, based on their location and conservation. The 10 SNPs are as follows: rs445678 in the promoter region; rs408753 in intron 3; un26537739, a novel synonymous (H107H); rs55906521 in intron 8; un26569149, a novel noncoding variant, 10 bp from a stop codon; rs10042, rs17055641, rs45471201 in the 3′-UTR; rs57045236, an 11 bp insertion/deletion, ~1 kb from 3′ of *DPYSL2*; and rs73229635, a noncoding variant, ~62 kb from 3′ of *DPYSL2*. The remaining two SNPs, rs17088251 and rs13277175, were included because of previous reports (Shifman *et al.* 2008; Liu *et al.* 2009). The SNP rs17088251, located ~7 kb 3′ of *DPYSL2*, has been associated with SZ risk in women (Shifman *et al.* 2008), whereas an association of rs13277175 (*DPYSL2* intron 8) with SZ has been reported in a CEU case-control study (Liu *et al.* 2009). We genotyped using Taqman custom SNP genotyping assays (Applied Biosystems) on the ABI 7900, according to the manufacturer’s recommendations. Among the selected SNPs, rs57045236, an 11-bp indel, was not amenable to assay by Taqman. Consequently, we amplified a 68-bp product encompassing this variant and scored the presence/absence of the 11-bp indel by standard agarose gel electrophoresis of the amplified products.

### Selection of regions for functional tests

To select regions for functional tests, we used 3 criteria: 1) contain one or more associated SNPs based on the sequencing and/or genotyping results; 2) meet criteria for a cNCR as defined previously; or 3) contain predicted CNS enhancers in a survey of the ~260 kb interval surrounding *DPYSL2* (Pennacchio *et al.* 2007). On the basis of these criteria, we chose four cNCRs for functional tests. Table S3 and Figure S2 provide detailed information.

### Dual luciferase assays (DLA)

#### HEK 293 cells:

We cultured HEK293 cells in Dulbecco’s modified Eagle’s medium supplemented with 10% fetal bovine serum and 1% penicillin and streptomycin in a humidified 5% CO_2_ atmosphere at 37°. Cells were transfected using Lipofectamine 2000 (Lipo2000, Invitrogen) according to the manufacturer’s instructions.

#### Primary mouse cortical neuronal cells:

We dissociated cells from E14.5 mouse cerebral cortices and seeded cells on poly-D-lysine−coated 24-well plates in plating medium (Opti-MEM containing 10% horse serum, 1% glutamax I, 1% penicillin-streptomycin, 1% N2 supplement, and 2.5M glucose). After 3 hr, we changed to maintenance medium (neurobasal medium containing 1% glutamax I, 2% B27 supplement, and 1% penicillin-streptomycin) and replaced half of maintenance medium with fresh maintenance medium every other day. All cultured neuronal populations comprised >99% neuronal cells as verified by immunostaining a sample of each dissociated population using chicken anti-MAP2 (see File S1).

#### pGL3 luciferase constructs:

For each of the four cNCRs selected for functional tests, we made two constructs in pGL3 vectors: one containing the nonassociated SNP allele, designated “LR” (low risk); the second, designated “HR” (high risk), containing the SZ- associated SNP allele (details provided in Table S3).

#### Transfection:

We used Lipo2000 to transiently transfect the pGL3 luciferase plasmid constructs containing *DPYSL2* sequence variants into 80–90% confluent HEK293 cells, or the third day *in vitro* cultured primary E14.5 cortical neurons. On the second day after transfection, we performed DLA (Invitrogen) according to the manufacturer’s protocol. For experiments with rapamycin (Selleckchem) exposure, cells were incubated with rapamycin at specified concentrations for 12 hr posttransfection. Cell lysis and dual luciferase assays continued immediately after the drug incubation period. We assayed six (HEK293) or 12 replicates (primary cortical neurons) for each construct in each experiment and repeated each experiment at least twice. We analyzed the data (12−24 readings for each construct) and generated figures using GraphPad Prism4.

#### Measurement of mRNA levels:

We transfected HEK293 cells at 80–90% confluence with constructs of DNR11, 12, 13, and 14. Twenty-four hours posttransfection, 10% of the cells were used for DLA following the procedure as described previously, and 90% of the cells were used to isolate RNA using RNeasy Mini Kit (QIAGEN). Synthesized cDNA via SuperScript III RT-PCR kit (Invitrogen) was used as the template to perform PCR with primers specific to the Luciferase transcript (F: AGAACTGCCTGCGTGAGATT; R: AAACCGTGATGGAATGGAA) for 25, 30, and 40 cycles. The PCR products were electrophoresed to separate amplicons for semiquantitative PCR analysis. We performed the same experiment in the absence of reverse transcriptase as a negative control measure to exclude contamination with luciferase from the plasmid DNA.

### Translation assays by polysome profiling

The location of rs3837184, a DNR in the 5′-UTR of *DPYSL2*, suggests that it could have an effect on translation efficiency. To test this possibility, we collected cell lysates from HEK293 transfected with two luciferase constructs, DNR11and 13. The cell lysates were prepared for polysome analysis and subjected to sucrose gradient size fractionation as previously described (Arava *et al.* 2003; Stefani *et al.* 2004). We extracted RNA from each of 12 fractions and used the same volume of each fraction to synthesize cDNA first-strands using SuperScript III RT-PCR kit (Invitrogen). The product was then analyzed by real-time reverse-transcription (RT)-PCR using luciferase gene-specific primers: F: AGAACTGCCTGCGTGAGATT; R: AAAACCGTGATGGAATGGAA. The calculated relative mRNA abundance was then used to determine the portion of mRNA found in each polysome fraction. Polysome β-actin mRNA abundance was measured in parallel as a negative control to ensure the correctness of the sample preparation. For details, see File S1.

### ZF transgenic reporter assays

#### pGW_cfosEGFP ZF reporter assay constructs:

We tested all four cNCRs for regulatory function by Tol2 transposon-mediated transgenic ZF reporter assay system (Fisher *et al.* 2006). As described in this published protocol, we amplified and cloned the four human cNCRs into the pCR8/GW/TOPO vector and then subcloned into pGW_cfosEGFP to get the final expression vector pCS_*cFos*EGFP. Function of the putative regulatory element was assessed by EGFP reporter gene expression *in vivo* (see Supplemental Notes).

#### Whole-mount in situ hybridization:

In ZF there is one annotated human *DPYSL2* ortholog (GenBank mRNA ID: NM_001020517, Ensembl mRNA ID: ENSDART00000064875), referred to herein as *dpysl2a*. Using a blastp search with human *DPYSL2* amino acid sequence and a subsequent blastn search of ESTs from ZF, we identified a second putative *DPYSL2* ortholog (GenBank mRNA ID: XR_084304; Ensembl mRNA ID: ENSDART00000056885) and refer to it as *dpysl2b*. ZF *dpysl2a* and *dpysl2b* paralogs are 87.1% identical to each other and 89.7% and 82.4% identical to human ortholog, respectively. We purchased the ZF *dpysl2a* EST clone CK148500 from Open biosystems. To make *dpysl2a* digoxygenin (DIG)-labeled antisense RNA probe, we digested CK148500 with *Spe* I and transcribed with T7 polymerase (Stratagene). We synthesized a DIG-labeled sense probe for *dpysl2a* with the SP6 polymerase (Stratagene) after *Acc* I digestion. To make *dpysl2b* DIG-labeled RNA probe, we extracted RNA from 48 hpf wild-type AB ZF and performed RT-PCR using Superscript III one-step RT-PCR kit (Invitrogen; primer sequences: F: CAT CTG GGA CAA GTG TGT GG; R: CCT TCT CTG TGG AGG GAC TG). The 652-bp PCR product was cloned into PCR2.1-TOPO vector (Invitrogen), its recombinant plasmids were linearized with *Hin*dIII, and the complementary RNA probe was synthesized using T7 polymerase with DIG labeling system. Whole-mount ISH on embryos at 24 hpf, 48 hpf, 72 hpf, and 96 hpf were performed using standard protocols. Images were acquired with SteREO Lumar.V12 microscope (Zeiss).

#### ZF maintenance:

ZF were maintained and bred under standard conditions at 28.5° (Westerfield 2000). Embryos were staged and fixed at specific hours post-fertilization (hpf) as described elsewhere (Kimmel *et al.* 1995). To better visualize *in situ* hybridization results, embryos were grown in 0.2 mM 1-phenyl-2-thiourea (Sigma) to inhibit pigment formation (Westerfield 2000).

### *DPYSL2* expression in brain tissues

We obtained 190 postmortem samples from the temporal lobe (Brodmann area 22) of donors without pathology from the Harvard Brain Bank. Characteristics of the samples (age, sex, and postmortem interval distribution) are provided in Table S4 and Figure S3.

#### Sample preparation and real-time PCR:

RNA and DNA were isolated from temporal lobe samples using AllPrep DNA/RNA Mini Kit (QIAGEN). We genotyped the DNAs for three SNPs (rs367948, rs400181, rs445678) in the promoter region, the DNR (rs3837184) in the 5′-UTR, and one SNP (rs73229635) in the 3′- flanking of *DPYSL2* by standard Sanger sequencing. We used the RNAs for generating first-strand cDNAs using Taqman Reverse Transcription Reagent and the resulting cDNAs for real time PCRs using *DPYSL2* transcript-specific primers (Table S5) following manufacturer’s protocols (ABI, SYBR Green PCR master mix) on ABI 7900 system. Expression of *DPYSL2* was normalized to the average expression level of two constitutively expressed genes: *ACTB* and *MRIP*. All samples were assayed in triplicate and quantified against a curve of standard dilutions, also in triplicate. The resulting measurements were normalized as described previously and then log-transformed to achieve a normal distribution for further statistical testing. The gene expression data were then analyzed using generalized linear/non-linear models in the STATISTICA version 7.1 software package (StatSoft Inc.; www.statsoft.com).

### Statistical analysis

We performed case-control association analyses using the likelihood ratio−based χ^2^ test implemented in the UNPHASED computer program v3.0.9 (Kimmel *et al.* 1995) and calculated haplotype frequencies, the LD coefficients (D′), and correlation coefficients (r^2^) with Haploview software (Barrett *et al.* 2005). To determine the statistical significance of differences between allelic or genotypic frequencies of variants from our sequencing results, we performed the Fisher exact test due to the small sample sizes. We used logistic regression analysis to determine the contribution of SNPs in the two haplotypes to the risk for SZ using STATISTICA 9.0.

## Results

Using PCR-based Sanger sequencing, we surveyed a total of 36.9 kb (6.7 kb of coding and 30.2 kb of noncoding sequence/individual) in 137 SZ probands (89 AJ and 48 CEU) and 151 controls (55 AJ and 96 CEU), generating a total of 4.1 Mb of high-quality sequence data (Table 1 and Figure S1). We identified a total of 120 variants, of which 10 (8%) were not listed in dbSNP (build 135 hg19; Table S6).

**Table 1:** Summary of sequencing results

Measure	Value
No. of exons	14
Genomic sequence covered (kb)/individual:	
Total	33.5
Coding	6.3
Noncoding	27.2
Sequence overall, kb:	
Total	4082
Coding	1822
Noncoding	2260
No. of variants	
Total	120
Nonsynonymous	1
Synonymous	10
Noncoding	109
No. of known dbSNP covered	100
No. of dbSNP not discovered	10

Of the 11 coding variants, 10 were synonymous (nine known, one novel) and one was a rare missense variant (MAF = 0.007), rs148064770 (p.A267S), present as a heterozygous allele in one of the CEU SZ probands. CRMP2B-A267 is conserved across the vertebrate linage and the variant (A267S) is predicted to be damaging by SIFT (Yuasa-Kawada *et al.* 2003) but benign by PolyPhen (Sunyaev *et al.* 2000, 2001; Ramensky *et al.* 2002).

### Variants of interest identified in the CEU

Among the 109 identified noncoding variants, six showed a statistically significant association with SZ in the CEU sample. All six lie at the 5′ end of *DPYSL2B* gene and include three SNPs in the proximal promoter in near complete LD with each other (rs367948, rs400181, rs445678; r^2^ = 0.97, minimum *P* = 0.007); a DNR in the 5′-UTR (rs3837184, *P* = 0.0003); and two SNPs in intron 1 (rs379266, *P* = 0.026; rs11781865, *P* = 0.017).

### Follow-up of identified variants by genotyping in the AJ cohort

**Figure 1:**
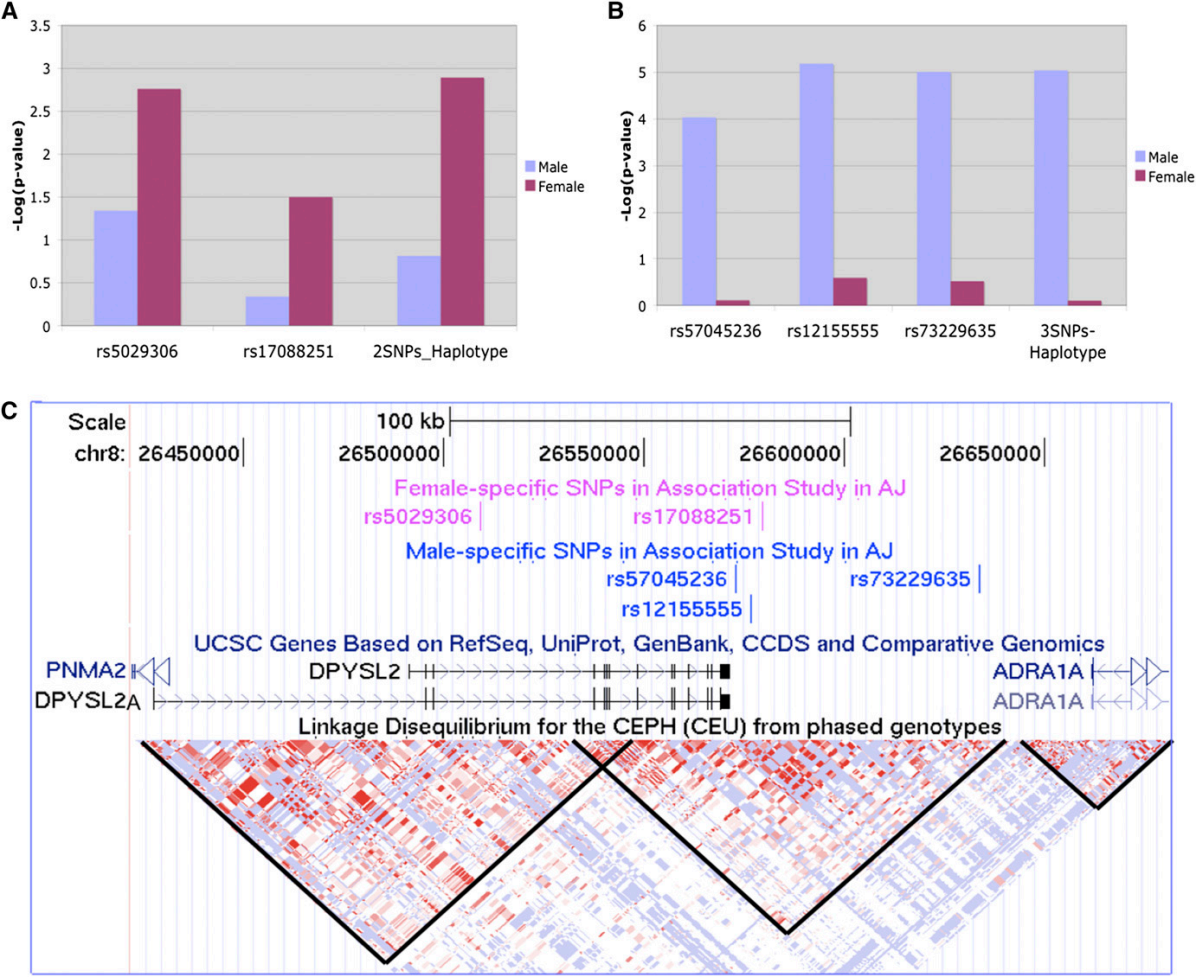
Identification of two sex-specific haplotypes with SZ susceptibility in 729 AJ SZ and 1542 AJ controls. (A) A 2-SNP (rs5029306, rs17088251) haplotype for female- specific SZ risk in AJ. (B) A 3-SNP (rs57045236, rs12155555, rs73229635) haplotype for male-specific SZ risk in AJ. (C) The same five SNPs shown in the UCSC genome browser (genome.ucsc.edu). The LD blocks, indicated by black lines, show that the SZ associated SNPs are in LD with *DPYSL2* but not *ADRA1A*. AJ, Ashkenazi Jewish; LD, linkage disequilibrium.

Of the SNPs selected for genotyping in the AJ cohort (see the section *Materials and Methods*) four ultimately failed to genotype due to poor clustering of assayed alleles. Five SNPs showed no statistically significant difference in allele frequency between cases and controls, or any effect on any of the nine phenotypic factors identified by principle component analysis as described in McGrath *et al.* 2009 (McGrath *et al.* 2009). Of the remaining SNPs, rs17088251 showed a case-control difference in females only, (*P* < 0.05). Similar results for rs17088251 were reported by Shifman *et al.* (2008), where it is among the top 1000 most statistically significant variants in AJ females in a study of 500,000 SNPs. SNP rs5029306 in intron 3, also showed a strong female-specific association alone (*P* = 0.0017) and in a haplotype with rs17088251 (*P* = 0.0013) (Figure 1A). These two SNPs are not in LD (r^2^ = 0.01); therefore, each is an independent predictor of the phenotype. The odds ratio for the risk haplotype in females is 2.15 (*P* = 0.001286) whereas in males it is 1.01 (*P* = n.s.). Finally, three variants in the ~90 kb gene-free region 3′ of *DPYSL2*, rs12155555 (ranking first in our 8p AJ study), rs57045236 and rs73229635, showed a significant association specific for males both individually (*P* = 6.6 × 10^−6^, *P* = 9.9 × 10^−6^, *P* = 9.2 × 10^−6^, respectively), and as a three SNP haplotype (*P* = 9.2 × 10^−6^) (Figure 1B). In males, the odds ratio for the risk haplotype is 2.04 compared with 1.22 in females. The pairwise r^2^ values of these three SNPs in the male-specific haplotype are 0.36, 0.19, and 0.5, respectively. In a multiple regression model only rs12155555 remained significant; thus, all three SNPs appear to reflect the same association signal.

### Disease-associated variants disrupt regulatory sequences

**Figure 2:**
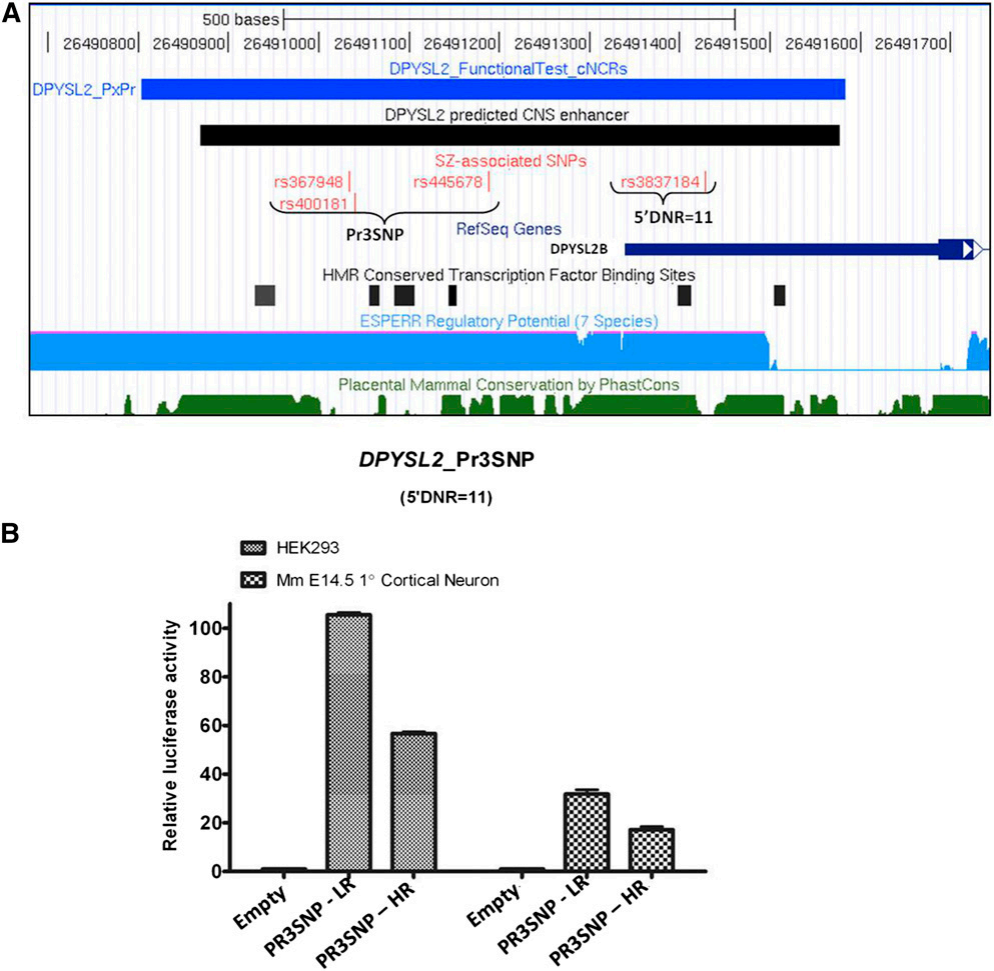
*DPYSL2* Pr3SNP luciferase constructs containing low risk (LR) *vs.* high risk (HR) haplotypes drive different levels of reporter expression *in vitro*. (A) Schematic representation of the human *DPYSL2* promoter region encompassing the 3-SNP haplotype (LR or HR) and a dinucleotide repeat (DNR) of fixed size (n = 11 repeats) (blue bar). (B) HR haplotype-containing constructs displayed lower luciferase expression than their LR counterparts when assayed in both cell types (HEK293 and primary cortical neurons). Each bar on the chart represents data derived from 6 technical replicates (HEK293) or 12 technical replicates (primary cortical neurons) and is consistent across two independent biological replicates. Error bar: standard error of the mean

Variants from those identified as described above to show associations with SZ, meeting criteria for a cNCR, and containing predicted CNS enhancers (see the section *Materials and Methods*) were selected for functional follow-up (Table 2). We selected four cNCRs that encompassed SZ associated SNPs for functional luciferase assays in neuronal (primary cortical) and non-neuronal (HEK293) cell types to determine the regulatory capacity of these sequences. We made two distinct reporter constructs designated Pr3SNP_LR (low risk) and Pr3SNP_HR (high risk) differing only in the three SNP promoter haplotypes. These included the same 782-bp insert extending 535 bp upstream of the transcription start site of *DPYSL2B* and 247 bp in the 5′-UTR (chr8: 26490803-26491584; hg18). Both carried the common number of repeats (11) for DNR rs3837184 (Figure 2A). The construct Pr3SNP_HR, which carried the high-risk haplotype, showed approximately a twofold decrease in luciferase activity in both primary E14.5 cortical neurons and HEK293 cells (*P* < 0.0001) (Figure 2B).

**Table 2:** Variants selected for functional assays

SNP	Location in *DPYSL2*	Functional Assay(s)
rs367948	Proximal promoter complete LD r^2^ ≥ 0.97	DLA, ZF transgenesis
rs400181		
rs445678		
rs3837184	5′ UTR DNR	DLA
rs379266	5′ intron 1	DLA, ZF transgenesis
rs11781865		
rs73229635	3′ of DPYSL2	DLA

DLA, dual luciferase assay; DNR, dinucleotide repeat; SNP, single-nucleotide polymorphism; UTR, untranslated region; ZF, zebrafish.

**Figure 3:**
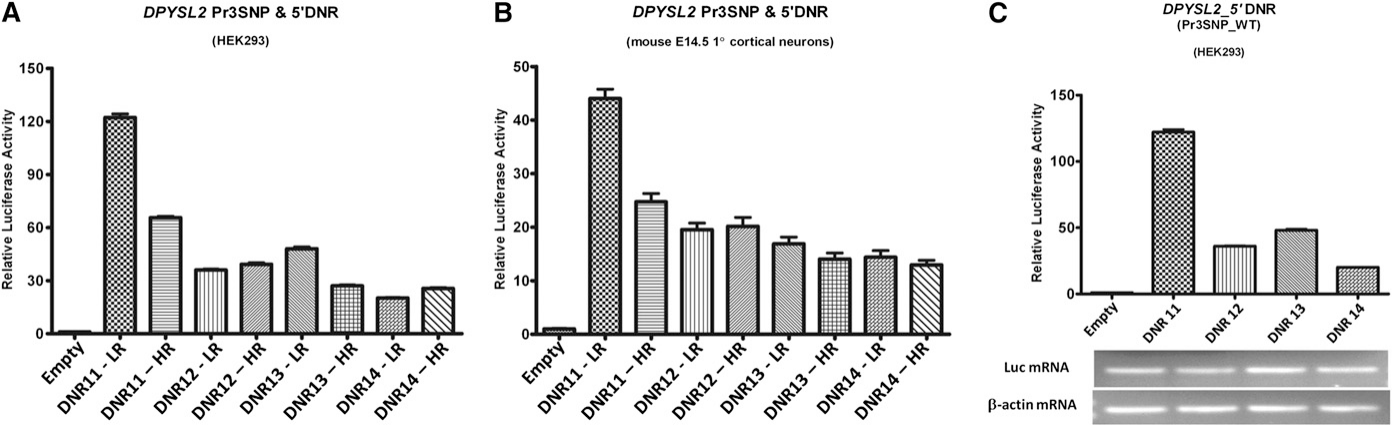
A dinucleotide repeat (DNR) in the *DPYSL2* 5′-UTR influences gene expression in *in vitro* reporter assays: Luciferase results from series of constructs containing LR/HR 3-SNP haplotype plus various DNR in HEK293 cells (A) and primary cortical neurons.* Risk 3-SNP haplotype with 13 DNRs (most common allele in DNR risk alleles) showed ~1.5- to 2-fold decrease in expression compared with construct having Risk 3-SNP haplotype with 11 DNRs in HEK293 (B) and primary cortical neurons (C). (D) these data were verified in HEK293 using semiquantitative expression level of transfected luciferase and endogenous Beta-actin mRNA were determined by reverse-transcription polymerase chain reaction (25 cycles).

To test for possible functional consequences of variation in the DNR (rs3837184), we made a series of constructs with the exact same sequence as the three SNP promoter haplotype, each containing DNR alleles of 11−14 repeats. After transfection into either HEK293 cells or primary E14.5 cortical mouse neurons, we observed a mean of ~3.5-fold decrease in luciferase expression with the high-risk alleles (DNR > 11, *i.e.*, 12, 13, or 14 repeats) compared with the low risk allele (DNR = 11 repeats) (*P* < 0.0001). The most common high-risk allele (DNR = 13 repeats) showed a fivefold decrease in expression in HEK293 cells (Figure 3, A and B). To explain this effect, we quantified the level of the chimeric 5′ DNR/luciferase mRNA in the transfected cells using semiquantitative RT-PCR and found similar amounts of chimeric mRNA for all variants (Figure 3C). In the absence of reverse transcriptase, we did not detect any product indicating that the assay is free of luciferase DNA sequence contamination and, therefore, reflects luciferase mRNA amount (Figure S4). We consider this a suggestion that the decrease in expression might be at the level of translation rather than transcription, which we followed up by polysome profiling below.

We also tested the intron 1 variants (rs379266, rs11781865) located in two different cNCRs in intron 1 (I1-1, I1-2; Table S3) of *DPYSL2A* or *B*. Both of these cNCRs had enhancer activity (expression increased ≥ 5 fold over basal levels) but neither showed altered expression between low risk and high risk alleles (Figure S5).

Finally, we tested rs73229635 in the intergenic region ~60 kb downstream of the 3′ end of *DPYSL2* and found a modest decrease in expression for the high-risk allele (cNCR_HR) in both primary E14.5 cortical neurons (~1.8 fold, *P* < 0.0001) and HEK293 cells (~1.2 fold, *P* < 0.0001) (Figure S6).

### ZF transgenesis reveals the biological relevance of implicated *DPYSL2* noncoding sequences

**Figure 4:**
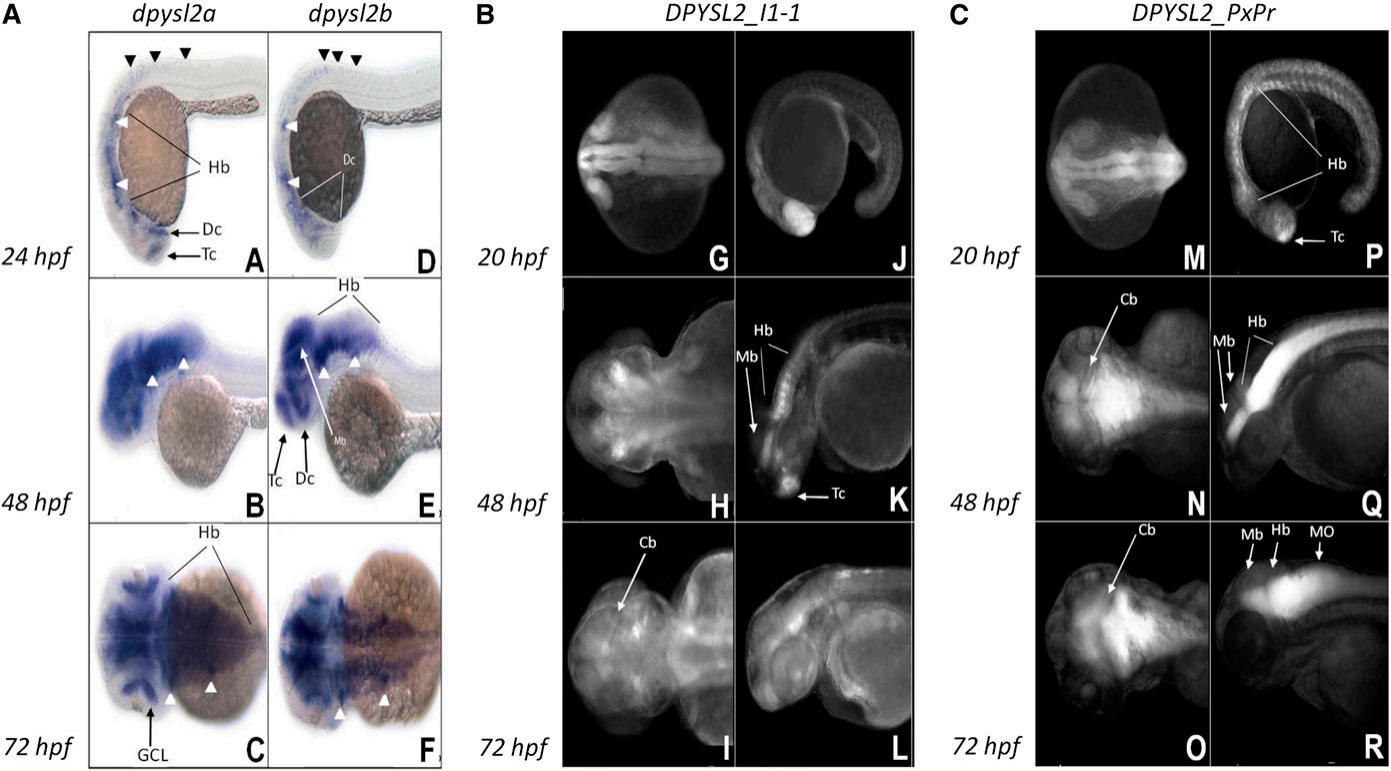
Expression pattern of ZF dpysl2 genes and DPYSL2_I1-1and DPYSL2_ShortProm elements in transgenic ZF. (A): A, B, C, D, E, F: dpysl2a and dpysl2b whole-mount *in situ* hybridizations at 24, 48, and 72 hpf. (B): G, H, I, J, K, L: Transgenic ZF line for DPYSL2_I1-1 element driving EGFP reporter expression at 20, 48 and 72 hpf. (C): M, N, O, P, Q, R: Transgenic ZF line for *DPYSL2_*ShortProm element driving *EGFP* expression at 20, 48, and 72 hpf; Cb, cerebellum; Dc, diencephalon; GCL, retinal ganglion cell layer; Hb, hindbrain; Mb, midbrain; MO, medulla oblongata; Tc, telencephalon. White arrowheads, anterior and posterior lateral line; black arrowheads, Rohon-Beard neurons; anterior is to the left. A, B, D, E, J, K, L, P, Q, and R: lateral view; C, F, G, H, I, M, N, and O: dorsal view.

We next used transgenic enhancer assay in ZF to assess the regulatory potential of the cNCRs containing the associated variants. We expect that sequences function as promoters/enhancers of human *DPYSL2* gene were likely to drive reporter EGFP expression in ZF in a pattern largely consistent with the endogenous *dpysl2* orthologs. First, we assayed the expression of the *dpysl2* ZF genes by *in situ* hybridization in wild-type ZF embryos at 24, 48, 72, and 96 hr postfertilization (hpf) (Figure 4, A−F, Figure S7). The pattern of spatial and temporal expression of *dpysl2a and dpysl2b* genes is largely overlapping, although not identical. At 24 hpf, *dpysl2a* transcript is present in the telencephalic and ventral diencephalic neuronal clusters, (Figure 4A and Figure S5, A and B), whereas *dpysl2b* gene is expressed in the ventral and dorsal diencephalon and midbrain areas (Figure 4D and Figure S5, C and D). Both transcripts also are present in the hindbrain, anterior and posterior lateral line (*anterior lateral line* and *posterior lateral line*, white arrowheads in Figure 4, A−C) placodes, and primary Rohon-Beard neurons (Figure 4A, D and Figure S5, A-D).

At 48 hpf, as the brain develops and expands, we observed more abundant expression of both genes. Although *dpysl2a* telencephalic expression fades away, there is a clear induction of *dpysl2b* transcription in this region at that stage. Both transcripts are present in the diencephalon, midbrain tectum, and tegmentum regions, as well as in the all hindbrain rhombomeres. Both genes also are expressed in the retinal ganglion cell layer. The *dpysl2a* and *dpysl2b* genes remain expressed in *anterior lateral line* and *posterior lateral line* ganglia and diffuse expression of both transcripts is observed in the spinal cord (Figure 4, B−F and Figure S5, E−H).

At 72 and 96 hpf, expression becomes weaker and is confined to the anterior CNS (Figure S5, I−P). Both transcripts are rather ubiquitously expressed in all brain regions, including ganglion and internal nuclear retinal layers and cranial ganglia (Supplemental Figure S5, I−P).

We generated four constructs of pGW-cfosEGFP for ZF transgenesis using the same human sequences tested in the cellular reporter assays (Table S3). Constructs containing *DPYSL2*_I1-1 and *DPYSL2*_PxPr drove tissue-specific expression in the CNS and PNS in both G0 mosaic and F1 progeny (Figure 4, G−R, Figure S8).

The *DPYSL2B*_I1-1 element directed *EGFP* expression in a pattern largely overlapping endogenous *dpysl2* expression (Figure 4, G−L). At early stages the transgene was strongly expressed in the telencephalon, diencephalon, and developing eye and moderately in the hindbrain region (Figure 4, G and J). Later, at 48 and 72 hpf, strong *EGFP* expression was maintained in the telencephalon and hindbrain rhombomeres, with weaker expression in the midbrain tegmentum, dorsal diencephalon, medulla oblongata and the spinal cord (Figure 4, H, I, K, and L).

The *DPYSL2B*_PxPr construct contained the most highly associated haplotype in the *DPYSL2* promoter region and directed transgene expression from the early stages of ZF development, where it was most abundant in the telencephalon and hindbrain regions (Figure 4, M and P). At later stages, expression of *EGFP* was restricted to but equally strong in the midbrain tegmentum, cerebellum, remaining hindbrain rhombomeres, and the spinal cord (Figure 4, N, O, Q, and R). In particular, this spatial and temporal pattern of expression is highly similar to the one driven by the *DPYSL2B* I1-1 element and resembles endogenous *dpysl2a* expression in the telencephalic and hindbrain regions.

Thus, our data demonstrate that these human sequences display regulatory function in ZF CNS in a pattern similar to the orthologous ZF genes. Further, this observation is consistent with our hypothesis that the human disease- associated variants in these sequences contribute risk for SZ by altering *DPYSL2B* expression.

### *DPYSL2* gene expression correlates with genotypes of five SZ-associated SNPs

To relate our cultured cell and ZF results to *in vivo* human expression, we examined *DPYSL2* expression in mRNA isolated from human brain. We designed forward primers specific for the alternative first exons of *DPYSL2* and a common reverse primer (DPYSL2A-F, DPYSL2B-F, and DPYSL2-R; Table S5). We confirmed expression of the transcripts by sequencing amplicons from temporal lobe cDNA pooled from multiple individuals. We then used these primer pairs for transcript-specific real-time PCR amplification of RNA harvested from the temporal lobe of 190 human brains without gross pathology (controls) obtained from the Harvard Brain Bank. To determine whether the genotype of selected SNPs correlated with mRNA expression, we genotyped five variants (SNPs rs367948, rs400181, and rs445678 in the promoter region; the DNR, rs3837184, in the 5′-UTR of *DPYSL2B*; and rs73229635 in the 3′-flank) in genomic DNAs isolated from these same 190 brain samples.

Expression of the *DPYSL2A and B* transcripts was strongly positively correlated (*P* < 10^−5^). In a generalized linear model that included age, sex, postmortem interval, and the alternative *DPYSL2* transcript we evaluated the SNP variants as predictors of *DPYSL2A* and *B* expression. *DPYSL2*B expression was correlated with the three SNP promoter haplotype (high-risk haplotype greater expression, *P* = 0.033). There was no correlation with sex. The direction of this correlation was opposite to that in our *in vitro* luciferase assay in which high-risk haplotype showed decreased expression. *DPYSL2A* expression was correlated with sex (higher expression in males, *P* = 0.017), age (higher expression in younger people, *P* < 10^−4^) and a sex*age interaction (expression decreased faster in males than in females with age, *P* = 0.0065). We also found correlation between *DPYSL2*A expression and SNP rs73229635 in the 3′ flank *DPYSL2* (high-risk allele greater expression, *P* = 0.013) and an interaction between rs73229635 and rs3837184 (DNR) with the presence of the high-risk alleles leading to lower expression in both cases (*P* = 0.008). Taken together, these *in vivo* results strongly support a role in the regulation of *DPYSL2* expression by the same variants implicated by our results in cultured cell systems.

### The polymorphic DNR in the *DPYSL2B* 5′-UTR influences translation via the mTOR pathway

**Figure 5:**
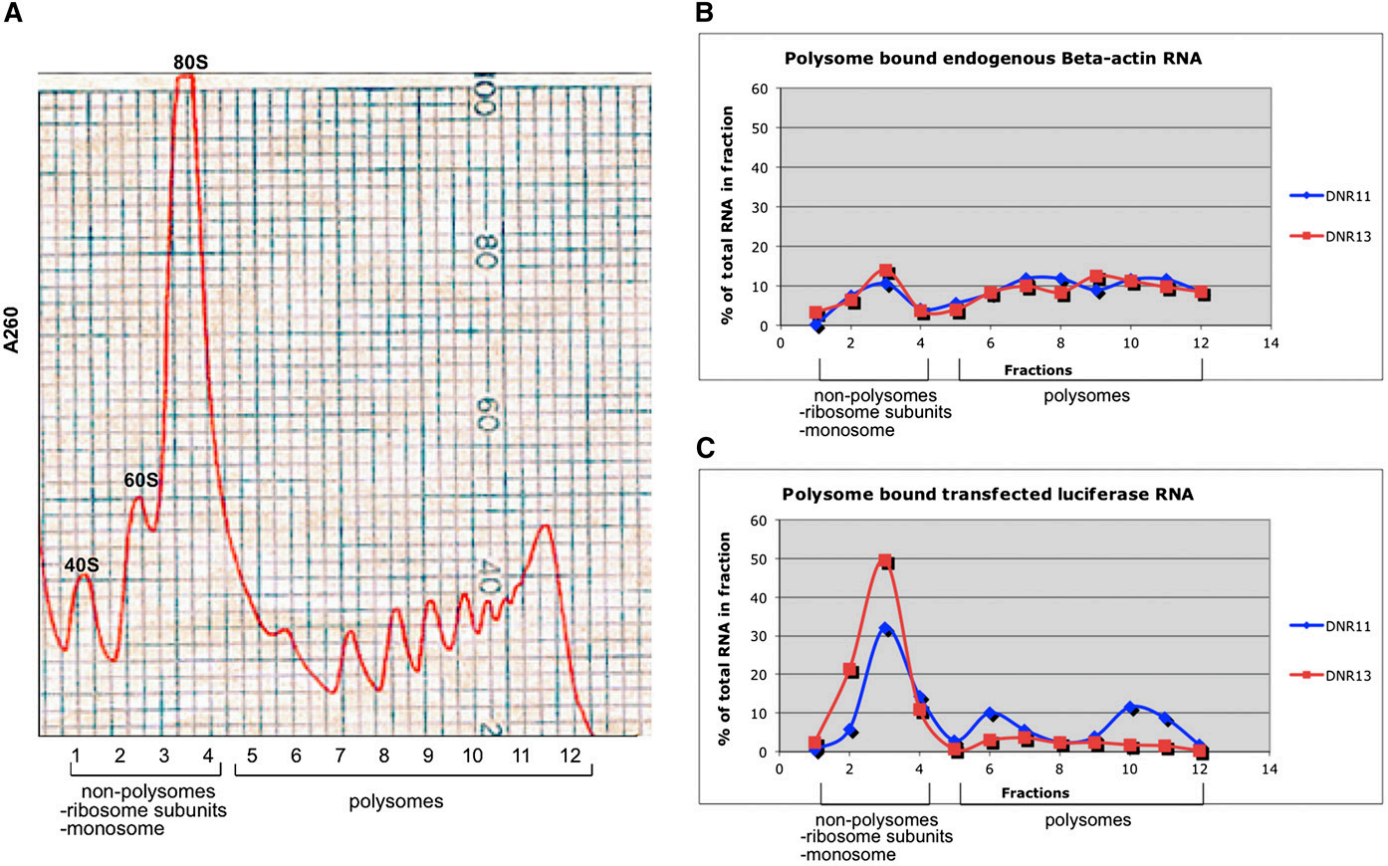
(A) Representative polysome profile of the 260-nm ultraviolet absorption through the sucrose gradient. Cytoplasmic extracts from HEK293 cells transfected with DNR luciferase constructs were layered over a 15–50% sucrose gradient. A total of 12 fractions (850 μL) were collected from the top of the gradient, and absorbance was measured at 260 nm to identify fractions containing monosomes and polysomes. Fraction 1 was devoid of any ribosomes, and fraction 2s−4 were nonpolysomes containing ribosome subunits and monosomes. Polysomes were present in fractions 5−12. (B and C) Distribution of endogenous beta-actin mRNA and exogenous luciferase mRNA in polysomes separated into 12 fractions by sucrose gradient. Blue line: cell lysates from HEK293 cells transfected with DNR 11 repeats; red line: HEK293 cell lysates from HEK293 cells transfected with DNR 13 repeats. (B) Endogenous beta-actin mRNA was found mostly on polysome fractions in both samples indicating active translation. (C) Polysome distribution of luciferase mRNA generated from transfected cells differed between DNR11 and DNR13—with more mRNA on polysomal fractions from DNR11 (47%) than DNR13 (15%).

Our cellular assays showed a 3.5- to 50-fold reduction in luciferase expression with the longer high-risk alleles (DNR ≥ 11) but no significant change in luciferase mRNA, suggesting a possible role of the DNR at the level of translation. We therefore analyzed the polysome profiles in HEK293 cells transfected with constructs of DNR11 and DNR13 and measured OD to ensure that the global distribution of polysomes was similar in all samples (Figure 5A). We collected 12 sucrose gradient fractions of the polyribosome preparations from cells transfected with each construct; each fraction containing mRNAs with different degrees of ribosome occupancy. We performed RT-quantitative PCR to measure the relative amount of luciferase mRNA in each fraction and also β- actin transcripts to control for any biases in harvesting mRNA across the gradients (Figure 5, B and C). The results are shown in Figure 5C. The Y-axis represents % of total actin or luciferase RNA. The actin fractions show little variation particularly across polysomes with most fractions containing ~10% of the RNA. In contrast smaller portions of luciferase RNA are seen in polysome fractions and importantly the portion of the DNR13 associated mRNA is threefold lower as compared with DNR11 (15% of total compared to 47% of total).

**Figure 6:**
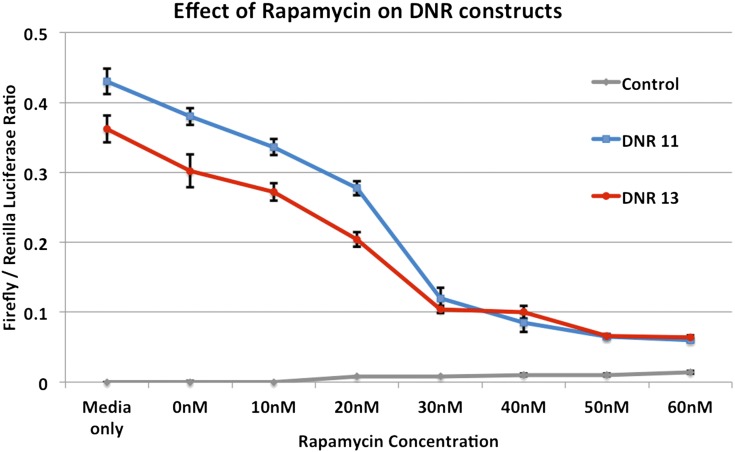
Effect of the mTOR inhibitor rapamycin on the DNR-driven expression of luciferase in HEK293 cells. The effects on the empty vector and the two 11 (WT) and 13 (Risk) DNR alleles are shown for a range of rapamycin concentrations. Rapamycin decreases luciferase activity for both alleles and reaches a plateau after ~30 nM. At this concentration the difference between the two alleles is no longer significant.

Further analysis of this DNR revealed that it contains a 5′-terminal oligopyrimindine (5′-TOP) tract, a hallmark of genes regulated by mTOR signaling. To explore how regulation of *DPYSL2* expression may be influenced by mTOR signaling, we performed dual luciferase assays with HEK293 transfected with either the low-risk allele (DNR = 11) or the high-risk allele (DNR = 13) in the absence or presence of mTOR inhibitors. Our results show that in the presence of increasing concentrations of rapamycin, an allosteric mTOR inhibitor, the high risk allele (DNR = 13) shows decreased expression compared to the low risk allele (DNR = 11) at concentrations ranging from 0-30 nM. At concentrations greater than 30nM both alleles reached a plateau at the same low level (Figure 6), suggesting complete inhibition of mTOR signaling. We showed the same response trend in mouse primary cortical neurons. From this we concluded that the presence of two additional di-nucleotide repeats in the 5′-UTR of *DPYSL2* results in a substantial reduction in the fraction of the mRNA in the polysomes, a reduction likely mediated by mTOR signaling.

## Discussion

We have followed up association signals by deep sequencing, molecular studies, functional assays, and studies of steady-state human brain mRNA levels to investigate the possible contribution of *DPYSL2* variants to risk for SZ. In addition to confirming the expression of two splice forms for *DPYSL2* and identifying multiple elements that regulate *DPYSL2* expression, we found several variants associated with risk for SZ, including three SNPs in the proximal promoter, two SNPs in intron 1 of *DPYSL2B*, and two SNP haplotypes over the body and 3′flanking sequence *of DPYSL2* that confer sex-specific risk for SZ. Moreover, we show that a SZ-associated polymorphic DNR in the 5′-UTR decreases *DPYSL2B* mRNA translation, likely through a modification of the response to mTOR signaling.

The six noncoding variants we identified in the 5′ region of *DPYSL2* showed associations with the risk for SZ only in the admittedly small CEU sequencing sample, yet their location (promoter region, 5′ UTR, and intron 1 NCRs) suggested possible effects on *DPYSL2* expression regulation. Consistent with these data, both cell-based and transgenic ZF assays clearly showed biologically relevant enhancer function of the cNCR I1-1 encompassing rs379266. The construct containing I1-1 directs reporter expression *in vivo* that largely recapitulates the endogenous patterns of *dpysl2* expression. Despite the genetic evidence implicating these sequences and their biologically pertinent *in vivo* behavior we observed no significant quantitative difference in the *in vitro* activity displayed by the alternative alleles of the SZ- associated SNP, rs379266 (Figure S4). There are several explanations for this negative result: 1) the *in vitro* assays are inherently synthetic and may not reflect the impact of a SNP in its endogenous chromatin context and 2) the associated SNP may impact spatial or temporal aspects of *DPYSL2* expression that may not be modeled well in this assay.

To determine whether the SZ associated DNA variants have *in vivo* effects, we looked for correlations between genotype and *DPYSL2* transcript levels in post mortem brain, specifically, in the superior temporal lobe. The same SNPs implicated by *in vitro* studies showed statistically significant correlations with the levels of *DPYSL2* mRNA *in vivo* indicating the effects of these variants can be discerned in brain samples. The direction of the effect comparing the *in vitro* and *in vivo* experiments was not always consistent. This may reflect the difference between *in vitro* experiments, which test a single variant in an artificial context, compared with *in vivo*, which reflects a snapshot of the summation of all regulatory effects in the context of a diverse population of cells from a specific brain region. In our view, more important than the direction of these differences is the fact that they exist. It is intriguing that we consistently find functional consequences for these SZ-associated variants across multiple study approaches.

We identified two haplotypes that confer sex-specific risk for SZ in our AJ sample (Figure 1). In agreement with our results, one of the SNPs we tested, rs17088251, was among the top 0.06% SNPs ranked by *P*-value in a genome-wide association study involving ~500,000 SNPs in a large, independent AJ female sample (Shifman *et al.* 2008). Our association results are consistent with that study, and we were able to show a more significant effect with a haplotype analysis that included a second SNP, rs5029306, 70.5 kb upstream in intron 3. These two SNPs appear to represent independent association signals. This observation strengthens our conclusion that we have identified a haplotype in the body of *DPYSL2* that confers a female-specific risk for SZ with no discernible effect in males. Conversely, we also identified a 3 SNP haplotype in the 3′- flanking region of *DPYSL2* that confers risk for males but not for females. These 3 SNPs are in LD with each other and appear to represent a single association signal. This male-specific risk haplotype is in a LD block that does not include the downstream gene *ADRA1A* (Figure 1C).

We also tested functionally the cNCR (chr8:26490803-26491584; hg18) that covers the predicted proximal promoter of *DPYSL2B*, including the three identified promoter SNPs and the DNR in the 5′UTR. Once again we confirmed regulatory function for all four variants in both *in vitro* dual cellular luciferase assays (Figure 2 and Figure 3) and *in vivo* transgenic reporter assay in ZF (Figure 4, M−R). In our CEU sample, the promoter three SNP haplotype was highly correlated with the DNR (D′ = 0.97; r^2^ = 0.94). The luciferase assay results indicate that the DNR is the major variant in regulating gene expression. These results do not exclude the possibility that the three SNP promoter haplotype and the DNR regulate gene expression synergistically, one at the level of transcription and one at the level of translation influenced by mTOR signaling.

The DNR polymorphism had the most significant effect on *DPYSL2B* expression and involved translational rather than transcriptional regulation. RT-PCR results from transient transfected HEK293 cells shows that DNR variants have no effect on mRNA abundance (Figure 3C). The substantial reduction (threefold) in the polysomal fraction *of DPYSL2* mRNA transcribed from the SZ-associated risk allele (13 DNRs) is consistent with an effect on translation and is directly proportional to the extent of functional defect observed in the DLA (Figure 3). These data strongly support the hypothesis that the expression of *DPYSL2B* is controlled posttranscriptionally and the SZ risk allele of the polymorphic DNR in the 5′-UTR reduces the expression of CRMP2B. This is also supported by a recent study (Morita and Sobue 2009), which identified a 5′-TOP tract in the 5′-UTR of *Dpysl2*. Using rat as a model, the authors showed that the translation of *DPYSL2B* mRNA is localized in the axon and is regulated by mTOR through the action of its effector, 70-kDa ribosomal S6 protein kinase (p70S6K), leading to the specification, formation, and maintenance of the axon. The SZ-causative polymorphic DNR identified in the present study is located in the middle of this so-called 5′-TOP tract, which in humans contains a 21-bp insert that includes part of the polymorphic sequence (Figure S9). As is the case for other 5′-TOP transcripts (Thoreen *et al.* 2012), it is likely that human *DPYSL2* protein synthesis is also under the control of the mTOR-P70S6K pathway locally in the axon. Phosphorylation of ribosomal protein S6, the substrate of P70S6K, increases the affinity of ribosomes for 5′-TOP mRNAs and facilitates the translation initiation of this category of mRNAs (Jefferies *et al.* 1997). Thus, we speculated that binding of the phosphorylated ribosomal protein S6 to the human *DPYSL2* 5′-TOP tract is sensitive to the number of DNRs, with the 11 DNR transcript binding more efficiently than the 13 DNR transcript. Our results show that both alleles are sensitive to rapamycin, a potent mTOR inhibitor, in a dose-dependent manner up to a critical threshold, after which there are no more differences between the two alleles (Figure 6). The results with rapamycin are consistent with our speculations about the link to mTOR signaling, and we are currently investigating the possible targets of mTOR that bind to the 5′-UTR of *DPYSL2* to mediate the changes in gene expression that is observed between the two alleles. Given the crucial role of mTOR in sensing environmental variables such as growth factors, nutrients (amino acids), and hormones, we propose that the risk alleles of the DNR interfere with mTOR induced translation of *DPYSL2B* mRNA in the developing axon leading to decreased CRMP2B with resulting abnormal axonogenesis. We further speculate that the mTOR-regulated translation of *DPYSL2* mRNA could provide a potential link to environmental variables (low birth weight, ABO incompatibility and perinatal problems) that have been shown to confer risk for SZ (Insel *et al.* 2005; Abel *et al.* 2010; Indredavik *et al.* 2010).

In summary, we systematically studied a biologically relevant SZ candidate gene, *DPYSL2*, and demonstrated a strong association of two sex-specific risk haplotypes. We provide strong evidence that SZ-associated *DPYSL2* sequence variants have functional significance, including a 5′-UTR polymorphic DNR. Our data suggests that the length of the DNR perturbs a 5′-TOP sequence and influences translation efficiency as regulated by mTOR signaling.

## Supplementary Material

Supporting Information

## References

[bib1] Abel K. M. Wicks S. Susser E. S. Dalman C. Pedersen M. G., 2010 Birth weight, schizophrenia, and adult mental disorder: is risk confined to the smallest babies? Arch. Gen. Psychiatry 67: 923–930. 20819986 10.1001/archgenpsychiatry.2010.100

[bib2] Aleman A. Kahn R. S. Selten J. P., 2003 Sex differences in the risk of schizophrenia: evidence from meta-analysis. Arch. Gen. Psychiatry 60: 565–571. 12796219 10.1001/archpsyc.60.6.565

[bib3] Arava Y. Wang Y. Storey J. D. Liu C. L. Brown P. O., 2003 Genome-wide analysis of mRNA translation profiles in *Saccharomyces cerevisiae*. Proc. Natl. Acad. Sci. USA 100: 3889–3894. 12660367 10.1073/pnas.0635171100 PMC153018

[bib4] Arimura N. Menager C. Fukata Y. Kaibuchi K., 2004 Role of CRMP-2 in neuronal polarity. J. Neurobiol. 58: 34–47. 14598368 10.1002/neu.10269

[bib5] Barrett J. C. Fry B. Maller J. Daly M. J., 2005 Haploview: analysis and visualization of LD and haplotype maps. Bioinformatics 21: 263–265. 15297300 10.1093/bioinformatics/bth457

[bib6] Beardsley T., 1996 Vital data. Sci. Am. 274: 100–105. 8607013 10.1038/scientificamerican0396-100

[bib7] Beasley C. L. Pennington K. Behan A. Wait R. Dunn M. J., 2006 Proteomic analysis of the anterior cingulate cortex in the major psychiatric disorders: evidence for disease-associated changes. Proteomics 6: 3414–3425. 16637010 10.1002/pmic.200500069

[bib8] Bergen S. E. Petryshen T. L., 2012 Genome-wide association studies of schizophrenia: does bigger lead to better results? Curr. Opin. Psychiatry 25: 76–82. 22277805 10.1097/YCO.0b013e32835035dd PMC3771358

[bib9] Blouin J. L. Dombroski B. A. Nath S. K. Lasseter V. K. Wolyniec P. S., 1998 Schizophrenia susceptibility loci on chromosomes 13q32 and 8p21. Nat. Genet. 20: 70–73. 9731535 10.1038/1734

[bib10] Cardno A. G. Rijsdijk F. V. Sham P. C. Murray R. M. McGuffin P., 2002 A twin study of genetic relationships between psychotic symptoms. Am. J. Psychiatry 159: 539–545. 11925290 10.1176/appi.ajp.159.4.539

[bib11] Chen P. L. Avramopoulos D. Lasseter V. K. McGrath J. A. Fallin M. D., 2009 Fine mapping on chromosome 10q22-q23 implicates Neuregulin 3 in schizophrenia. Am. J. Hum. Genet. 84: 21–34. 19118813 10.1016/j.ajhg.2008.12.005 PMC2668048

[bib12] Chumakov I. Blumenfeld M. Guerassimenko O. Cavarec L. Palicio M., 2002 Genetic and physiological data implicating the new human gene G72 and the gene for D-amino acid oxidase in schizophrenia. Proc. Natl. Acad. Sci. USA 99: 13675–13680. 12364586 10.1073/pnas.182412499 PMC129739

[bib13] Eaton W. W., 1985 Epidemiology of schizophrenia. Epidemiol. Rev. 7: 105–126. 3902492 10.1093/oxfordjournals.epirev.a036278

[bib14] Edgar P. F. Douglas J. E. Cooper G. J. Dean B. Kydd R., 2000 Comparative proteome analysis of the hippocampus implicates chromosome 6q in schizophrenia. Mol Psych 5: 85–90. 10.1038/sj.mp.4000580 10673773

[bib15] Fallin M. D. Lasseter V. K. Wolyniec P. S. McGrath J. A. Nestadt G., 2003 Genomewide linkage scan for schizophrenia susceptibility loci among Ashkenazi Jewish families shows evidence of linkage on chromosome 10q22. Am. J. Hum. Genet. 73: 601–611. 12929083 10.1086/378158 PMC1180684

[bib16] Fallin M. D. Lasseter V. K. Avramopoulos D. Nicodemus K. K. Wolyniec P. S., 2005 Bipolar I disorder and schizophrenia: a 440-single-nucleotide polymorphism screen of 64 candidate genes among Ashkenazi Jewish case-parent trios. Am. J. Hum. Genet. 77: 918–936. 16380905 10.1086/497703 PMC1285177

[bib17] Fallin M. D. Lasseter V. K. Liu Y. Avramopoulos D. McGrath J., 2011 Linkage and association on 8p21.2-p21.1 in schizophrenia. Am. J. Med. Genet. B. Neuropsychiatr. Genet. 156: 188–197. 21302347 10.1002/ajmg.b.31154

[bib18] Fisher S. Grice E. A. Vinton R. M. Bessling S. L. Urasaki A., 2006 Evaluating the biological relevance of putative enhancers using Tol2 transposon-mediated transgenesis in zebrafish. Nat. Protoc. 1: 1297–1305. 17406414 10.1038/nprot.2006.230

[bib19] Foulkes W. D. Thiffault I. Gruber S. B. Horwitz M. Hamel N., 2002 The founder mutation MSH2*1906G→C is an important cause of hereditary nonpolyposis colorectal cancer in the Ashkenazi Jewish population. Am. J. Hum. Genet. 71: 1395–1412. 12454801 10.1086/345075 PMC420003

[bib20] Goldstein J. M. Faraone S. V. Chen W. J. Tolomiczencko G. S. Tsuang M. T., 1990 Sex differences in the familial transmission of schizophrenia. Br. J. Psychiatry 156: 819–826. 2207512 10.1192/bjp.156.6.819

[bib21] Häfner H., 2003 Gender differences in schizophrenia. Psychoneuroendocrinology 28(Suppl 2)**:** 17–54. 12650680 10.1016/s0306-4530(02)00125-7

[bib22] Häfner H. Maurer K. Löffler W. Riecher-Rössler A., 1993 The influence of age and sex on the onset and early course of schizophrenia. Br. J. Psychiatry 162: 80–86. 8425144 10.1192/bjp.162.1.80

[bib23] Holmans P. A. Riley B. Pulver A. E. Owen M. J. Wildenauer D. B., 2009 Genomewide linkage scan of schizophrenia in a large multicenter pedigree sample using single nucleotide polymorphisms. Mol. Psychiatry 14: 786–795. 19223858 10.1038/mp.2009.11 PMC2714870

[bib24] Inagaki N. Chihara K. Arimura N. Ménager C. Kawano Y., 2001 CRMP-2 induces axons in cultured hippocampal neurons. Nat. Neurosci. 4: 781–782. 11477421 10.1038/90476

[bib25] Indredavik M. S. Vik T. Evensen K. A. Skranes J. Taraldsen G., 2010 Perinatal risk and psychiatric outcome in adolescents born preterm with very low birth weight or term small for gestational age. J. Dev. Behav. Pediatr. 31: 286–294. 20431402 10.1097/DBP.0b013e3181d7b1d3

[bib26] Insel B. J. Brown A. S. Bresnahan M. A. Schaefer C. A. Susser E. S., 2005 Maternal-fetal blood incompatibility and the risk of schizophrenia in offspring. Schizophr. Res. 80: 331–342. 16006103 10.1016/j.schres.2005.06.005

[bib27] Insel T. R., 2010 Rethinking schizophrenia. Nature 468: 187–193. 21068826 10.1038/nature09552

[bib28] Jablensky A., 2006 Subtyping schizophrenia: implications for genetic research. Mol. Psychiatry 11: 815–836. 16801952 10.1038/sj.mp.4001857

[bib29] Jefferies H. B. J. Fumagalli S. Dennis P. B. Reinhard C. Pearson R. B., 1997 Rapamycin suppresses 5′TOP mRNA translation through inhibition of p70s6k. EMBO J. 16: 3693–3704. 9218810 10.1093/emboj/16.12.3693 PMC1169993

[bib30] Johnston-Wilson N. L. Sims C. D. Hofmann J.-P. Anderson L. Shore A. D.; The Stanley Neuropathology Consortium, 2000 Disease-specific alterations in frontal cortex brain proteins in schizophrenia, bipolar disorder, and major depressive disorder. Mol. Psychiatry 5: 142–149. 10822341 10.1038/sj.mp.4000696

[bib31] Kao W.-T. Wang Y. Kleinman J. E. Lipska B. K. Hyde T. M., 2010 Common genetic variation in Neuregulin 3 (NRG3) influences risk for schizophrenia and impacts NRG3 expression in human brain. Proc. Natl. Acad. Sci. USA 107: 15619–15624. 20713722 10.1073/pnas.1005410107 PMC2932571

[bib32] Kimmel C. B. Ballard W. W. Kimmel S. R. Ullmann B. Schilling T. F., 1995 Stages of embryonic development of the zebrafish. Dev. Dyn. 203: 253–310. 8589427 10.1002/aja.1002030302

[bib33] Li D. Collier D. A. He L., 2006 Meta-analysis shows strong positive association of the neuregulin 1 (NRG1) gene with schizophrenia. Hum. Mol. Genet. 15: 1995–2002. 16687441 10.1093/hmg/ddl122

[bib34] Liu Y. Chen P. L. Lasseter V. K. Fallin M. D. McGrath J., 2009 Variants in and around DPYSL2 confer sex-specific risks for schizophrenia. Am. J. Hum. Genet. 85: A871.

[bib35] Martins-de-Souza D. Gattaz W. F. Schmitt A. Maccarrone G. Hunyadi-Gulyás E., 2009 Proteomic analysis of dorsolateral prefrontal cortex indicates the involvement of cytoskeleton, oligodendrocyte, energy metabolism and new potential markers in schizophrenia. J. Psychiatr. Res. 43: 978–986. 19110265 10.1016/j.jpsychires.2008.11.006

[bib36] McGrath J. Saha S. Welham J. El Saadi O. MacCauley C., 2004 A systematic review of the incidence of schizophrenia: the distribution of rates and the influence of sex, urbanicity, migrant status and methodology. BMC Med. 2: 13. 15115547 10.1186/1741-7015-2-13 PMC421751

[bib37] McGrath J. Avramopoulos D. Lasseter K. Wolyniec P. Fallin M. D., 2009 Familiality of novel factorial dimensions of schizophrenia. Arch. Gen. Psych. 66: 591–600. 10.1001/archgenpsychiatry.2009.56 19487624

[bib38] Millar J. K. Wilson-Annan J. C. Anderson S. Christie S. Taylor M. S., 2000 Disruption of two novel genes by a translocation co-segregating with schizophrenia. Hum. Mol. Genet. 9: 1415–1423. 10814723 10.1093/hmg/9.9.1415

[bib39] Morar B. Dragović M. Waters F. A. Chandler D. Kalaydjieva L., 2011 Neuregulin 3 (NRG3) as a susceptibility gene in a schizophrenia subtype with florid delusions and relatively spared cognition. Mol. Psychiatry 16: 860–866. 20548296 10.1038/mp.2010.70

[bib40] Morita T. Sobue K., 2009 Specification of neuronal polarity regulated by local translation of CRMP2 and Tau via the mTOR-p70S6K pathway. J. Biol. Chem. 284: 27734–27745. 19648118 10.1074/jbc.M109.008177 PMC2785701

[bib41] Ng P. C. Murray S. S. Levy S. Venter J. C., 2009 An agenda for personalized medicine. Nature 461: 724–726. 19812653 10.1038/461724a

[bib42] Owen M. J. Craddock N. Jablensky A., 2007 The genetic deconstruction of psychosis. Schizophr. Bull. 33: 905–911. 17551090 10.1093/schbul/sbm053 PMC2632314

[bib43] Pennacchio L. A. Loots G. G. Nobrega M. A. Ovcharenko I., 2007 Predicting tissue-specific enhancers in the human genome. Genome Res. 17: 201–211. 17210927 10.1101/gr.5972507 PMC1781352

[bib44] Pulver A. E. Bale S. J., 1989 Availability of schizophrenic patients and their families for genetic linkage studies: findings from the Maryland epidemiology sample. Genet. Epidemiol. 6: 671–680. 2606340 10.1002/gepi.1370060604

[bib45] Pulver A. E. Lasseter V. K. Kasch L. Wolyniec P. Nestadt G., 1995 Schizophrenia: a genome scan targets chromosomes 3p and 8p as potential sites of susceptibility genes. Am. J. Med. Genet. 60: 252–260. 7573181 10.1002/ajmg.1320600316

[bib46] Pulver A. E. Mulle J. Nestadt G. Swartz K. L. Blouin J. L., 2000 Genetic heterogeneity in schizophrenia: stratification of genome scan data using co-segregating related phenotypes. Mol. Psychiatry 5: 650–653. 11126395 10.1038/sj.mp.4000814

[bib47] Ramensky V. Bork P. Sunyaev S., 2002 Human non-synonymous SNPs: server and survey. Nucleic Acids Res. 30: 3894–3900. 12202775 10.1093/nar/gkf493 PMC137415

[bib48] Rees E. Kirov G. O’Donovan M. C. Owen M. J., 2012 De novo mutation in schizophrenia. Schizophr. Bull. 38: 377–381. 22451492 10.1093/schbul/sbs047 PMC3329988

[bib49] Rice L. Handayani R. Cui Y. Medrano T. Samedi V., 2007 Soy isoflavones exert differential effects on androgen responsive genes in LNCaP human prostate cancer cells. J. Nutr. 137: 964–972. 17374662 10.1093/jn/137.4.964 PMC1975677

[bib50] Sawa A. Snyder S. H., 2002 Schizophrenia: diverse approaches to a complex disease. Science 296: 692–695. 11976442 10.1126/science.1070532

[bib51] Schizophrenia Psychiatric Genome-Wide Association Study (GWAS) Consortium, 2011 Genome-wide association study identifies five new schizophrenia loci. Nat. Genet. 43: 969–976. 21926974 10.1038/ng.940 PMC3303194

[bib52] Schizophrenia Working Group of the Psychiatric Genomics Consortium, 2014 Biological insights from 108 schizophrenia-associated genetic loci. Nature 511: 421–427. 25056061 10.1038/nature13595 PMC4112379

[bib53] Shifman S. Johannesson M. Bronstein M. Chen S. X. Collier D. A., 2008 Genome-wide association identifies a common variant in the reelin gene that increases the risk of schizophrenia only in women. PLoS Genet. 4: e28. 18282107 10.1371/journal.pgen.0040028 PMC2242812

[bib54] Shtasel D. L. Gur R. E. Gallacher F. Heimberg C. Gur R. C., 1992 Gender differences in the clinical expression of schizophrenia. Schizophr. Res. 7: 225–231. 1390401 10.1016/0920-9964(92)90016-x

[bib55] Siepel A. Bejerano G. Pedersen J. S. Hinrichs A. S. Hou M., 2005 Evolutionarily conserved elements in vertebrate, insect, worm, and yeast genomes. Genome Res. 15: 1034–1050. 16024819 10.1101/gr.3715005 PMC1182216

[bib56] St Clair D. Blackwood D. Muir W. Carothers A. Walker M., 1990 Association within a family of a balanced autosomal translocation with major mental illness. Lancet 336: 13–16. 1973210 10.1016/0140-6736(90)91520-k

[bib57] Stefani G. Fraser C. E. Darnell J. C. Darnell R. B., 2004 Fragile X mental retardation protein is associated with translating polyribosomes in neuronal cells. J. Neurosci. 24: 7272–7276. 15317853 10.1523/JNEUROSCI.2306-04.2004 PMC6729764

[bib58] Stefansson H. Sigurdsson E. Steinthorsdottir V. Bjornsdottir S. Sigmundsson T., 2002 *Neuregulin 1* and susceptibility to schizophrenia. Am. J. Hum. Genet. 71: 877–892. 12145742 10.1086/342734 PMC378543

[bib59] Straub R. E. Jiang Y. MacLean C. J. Ma Y. Webb B. T., 2002 Genetic variation in the 6p22.3 gene *DTNBP1*, the human ortholog of the mouse dysbindin gene, is associated with schizophrenia. Am. J. Hum. Genet. 71: 337–348. 12098102 10.1086/341750 PMC379166

[bib60] Sunyaev S. Ramensky V. Bork P., 2000 Towards a structural basis of human non-synonymous single nucleotide polymorphisms. Trends Genet. 16: 198–200. 10782110 10.1016/s0168-9525(00)01988-0

[bib61] Sunyaev S. Ramensky V. Koch I. Lathe W. 3rd Kondrashov A. S., 2001 Prediction of deleterious human alleles. Hum. Mol. Genet. 10: 591–597. 11230178 10.1093/hmg/10.6.591

[bib62] Szymanski S. Lieberman J. A. Alvir J. M. Mayerhoff D. Loebel A., 1995 Gender differences in onset of illness, treatment response, course, and biologic indexes in first-episode schizophrenic patients. Am. J. Psychiatry 152: 698–703. 7726309 10.1176/ajp.152.5.698

[bib63] Thoreen C. C. Chantranupong L. Keys H. R. Wang T. Gray N. S., 2012 A unifying model for mTORC1-mediated regulation of mRNA translation. Nature 485(7396): 109–113. 22552098 10.1038/nature11083 PMC3347774

[bib64] Xu B. Ionita-Laza I. Roos J. L. Boone B. Woodrick S. Sun Y. Levy S. Gogos J. A. Karayiorgou M., 2012 De novo gene mutations highlight patterns of genetic and neural complexity in schizophrenia. Nat. Genet. 44: 1365–1369. 23042115 10.1038/ng.2446 PMC3556813

[bib65] Yamashita N. Ohshima T. Nakamura F. Kolattukudy P. Honnorat J., 2012 Phosphorylation of CRMP2 (collapsin response mediator protein 2) is involved in proper dendritic field organization. J. Neurosci. 32: 1360–1365. 22279220 10.1523/JNEUROSCI.5563-11.2012 PMC6796265

[bib66] Yuasa-Kawada J. Suzuki R. Kano F. Ohkawara T. Murata M., 2003 Axonal morphogenesis controlled by antagonistic roles of two CRMP subtypes in microtubule organization. Eur. J. Neurosci. 17: 2329–2343. 12814366 10.1046/j.1460-9568.2003.02664.x

