# Supplementary material for: Functional Variants in DPYSL2 Sequence Increase Risk of Schizophrenia and Suggest a Link to mTOR Signaling
Source: G3 (Bethesda). 2014 Nov 20;5(1):61–72. doi: 10.1534/g3.114.015636 (PMC4291470; doi:10.1534/g3.114.015636)
Supplement: Supporting Information [file supp_5_1_61__index.html]

Functional Variants in DPYSL2 Sequence Increase Risk of Schizophrenia and Suggest a Link to mTOR Signaling — Supporting Information 

# Functional Variants in *DPYSL2* Sequence Increase Risk of Schizophrenia and Suggest a Link to mTOR Signaling

## Supporting Information for Liu *et al.*, 2015

**Files in this Data Supplement:**

- Supporting Information - File S1, Figures S1-S9, and Tables S1-S6 (PDF, 1 MB)
- File S1 - Supplemental Materials (PDF, 63 KB)
- Figure S1 - 27 cNCRs in and around *DPYSL2* shown as custom track (black bars) on UCSC genome browser, which covered virtually all the conserved regions in an ~260kb interval in and around *DPYSL2*. (PDF, 284 KB)
- Figure S2 - Four cNCRs selected for functional tests shown on UCSC genome browser in an ~260kb interval around *DPYSL2*. (PDF, 145 KB)
- Figure S3 - Distribution of PMI from 190 brain samples. (PDF, 61 KB)
- Figure S4 - Negative control of RT-PCR experiment with and without reverse transcriptase when amplifying luciferase from transfected cells. (PDF, 89 KB)
- Figure S5 - Human *DPYSL2* intron1 two constructs containing 2 SZ-associated SNPs did not show difference in driving luciferase expression between constructs containing Wt allele and Risk allele. (PDF, 179 KB)
- Figure S6 - Human *DPYSL2* 3'-flanking cNCR (3P-cNCR) construct containing a SZ-associated SNP rs73229635 (p-value<10-5 in large AJ collection) showed enhancer function and difference in driving luciferase expression between constructs containing Wt allele and Risk allele. (PDF, 133 KB)
- Figure S7 - Whole mount *in situ* hybridization with *dpysl2a* and *dpysl2b* riboprobes. (PDF, 89 KB)
- Figure S8 - Transgenic zebrafish *DPYSL2*\_PxPr line with *EGFP* reporter. (PDF, 191 KB)
- Figure S9 - Sequences of human and rat *DPYSL2* showing 5'-TOP sequence and polymorphic DNR in human sequence. (PDF, 119 KB)
- Table S1 - Sample counts for sequencing and genotyping. (PDF, 38 KB)
- Table S2 - Primers for *DPYSL2* 14 exons and 27 cNCRs. (PDF, 44 KB)
- Table S3 - Four cNCRs selected for functional tests. (PDF, 39 KB)
- Table S4 - Brain samples. (PDF, 38 KB)
- Table S5 - Primers used for real time PCR. (PDF, 38 KB)
- Table S6 - The list of 120 variants in and around *DPYSL2* identified by sequencing. (PDF, 44 KB)
