# Supplementary material for: Functional Variants in DPYSL2 Sequence Increase Risk of Schizophrenia and Suggest a Link to mTOR Signaling
Source: G3 (Bethesda). 2014 Nov 20;5(1):61–72. doi: 10.1534/g3.114.015636 (PMC4291470; doi:10.1534/g3.114.015636)
Supplement: Supporting Information [file supp_g3.114.015636_015636SI.pdf]

G3 · Genes | Genomes | Genetics

Functional Variants in *DPYSL2* Sequence Increase Schizophrenia Risk and Suggest a Link To mTOR Signaling

Yaping Liu *, §, Xuan Pham*, §, Lilei Zhang *, Pei-lung Chen *, § Grzegorz Burzynski * David M. McGaughey *, §, Shan He **, John A. McGrath §§, Paula Wolyniec §§, Margaret D. Fallin ***, Megan S. Pierce *, §, Andrew S. McCallion *, §§§, Ann E. Pulver §§, Dimitrios Avramopoulos *, David Valle, §

* McKusick-Nathans Institute of Genetic Medicine; § Predoctoral Training Program in Human Genetics; ** Department of Molecular Biology and Genetics; §§ Department of Psychiatry; *** Department of Epidemiology; §§§ Department of Molecular and
Comparative Pathobiology, Johns Hopkins University School of Medicine and, Johns

Hopkins Bloomberg School of Public Health, Baltimore, MD 21205

Corresponding Author: David Valle, M.D., Henry J. Knott Professor & Director, McKusick-Nathans Institute of Genetic Medicine, Johns Hopkins University School of Medicine, MRB 519, 733 N. Broadway, Baltimore MD 21205
dvalle@jhmi.edu

DOI: 10.1534/g3.114.015636

File S1

SUPPLEMENTAL MATERIALS

Study subjects

Our CEU multiplex families were recruited from multiple sources including our Maryland Epidemiologic Samples (MES) (Pulver and Bale 1989), our nationwide advertising efforts, and national and international collaborators (US, Italy, Poland, Greece). All families are of European-Caucasian ancestry. Descriptions of our clinical assessment methods are published elsewhere (Blouin *et al* 1998).

Our AJ SZ and controls were recruited in North America by advertisements in newspapers and Jewish newsletters, talks to community organizations, letters to leaders of the Jewish community, letters and talks to service providers and a study website hosted by the Johns Hopkins Epidemiology-Genetics Program (EpiGen) in Psychiatry (Fallin *et al* 2003). AJ SZ cases and controls self-identified Ashkenazi Jewish ethnicity in all four grandparents.

All CEU and AJ SZ cases met probable or definite DSM-IV schizophrenia or schizoaffective disorder criteria based on a consensus diagnosis. Our diagnostic methods are available in detail (Fallin *et al* 2005). All controls collected by the MES screened negative for a history of psychosis, mania, psychiatric hospitalization, depression or suicide attempts. All recruitment methods and protocols for collection of clinical data and blood samples were approved by the Johns Hopkins Institutional Review Board; informed consent was obtained from all subjects.

In the present study, we used an additional 721 controls of AJ descent from New York Cancer Project (NYCP) Biorepository (Foulkes *et al* 2002); the ethnicity of their grandparents is less certain.

Sequencing subjects

We selected 48 CEU SZ probands who were homozygous for the risk alleles for the SNP with high linkage signal. We sequenced 96 unrelated Centre d'Etude du Polymorphisme Humain (CEPH) samples as a CEU control. We chose 48 AJ SZ subjects from our sample because they have a positive family history for psychosis: i.e., at least two members of the family (the proband and one more first and second degree relatives) had evidence of psychosis either from direct assessment or from family informant data. The 48 AJ controls were all 40 year of age or greater (an age at which the risk of developing SZ is reduced by 90%) with no history of depression, mania, psychosis, or psychiatric hospitalization. We selected an additional 41 AJ SZ probands who were homozygous for the risk alleles at

both peak associated SNPs (rs12155555; rs5029306) and 7 AJ controls who were homozygous for the non-risk alleles at both rs12155555 and rs5029306 to identify variants segregating on the risk haplotype and to detect variants that might be specific to the 41 AJ SZ probands.

SNP selection and genotyping

The selected 10 SNPs are rs445678 in the promoter region, rs408753 in intron 3, un26537739, a novel synonymous (H107H), rs55906521 in intron 8, un26569149, a novel noncoding variant, 10bp from stop codon, rs10042, rs17055641, rs45471201 in the 3'-UTR, rs57045236, an 11bp insertion/deletion, ~1kb from 3' of *DPYSL2*, and finally rs73229635, a noncoding variant, ~62kb from 3' of *DPYSL2*.

HEK 293 cells culture

We cultured HEK293 cells in Dulbecco's modified Eagle's medium (DMEM) supplemented with 10% fetal bovine serum (FBS) and 1% penicillin and streptomycin in a humidified 5% CO_2 atmosphere at 37°C.

Primary embryonic day 14.5 (E14.5) mouse cortical neuronal cell culture

We dissociated cells from E14.5 mouse cerebral cortices and seeded cells on poly-D-lysine-coated 24-well plates in plating medium (Opti-MEM containing 10% horse serum, 1% glutamax I, 1% penicillin-streptomycin, 1% N2 supplement, and 2.5M glucose). After 3 hours, we changed to maintenance medium (neurobasal medium containing 1% glutamax I, 2% B27 supplement, and 1% penicillin-streptomycin) and replaced half of maintenance medium with fresh maintenance medium every other day.

Immunofluorescence staining

On the 3rd day of culture, we characterized the E14.5 mouse cortical neurons by immunostaining. Cells were fixed in 4% paraformaldehyde and permeabilized in 0.3% Triton and then incubated with anti-MAP2 (chicken polyclonal antibody, Abcam) and anti-GFAP (rabbit polyclonal antibody, Fisher Scientific) diluted at 1:100, followed by incubation with fluorescently labeled Goat anti-chicken/rabbit secondary antibodies (Molecular Probe) at a 1:500 dilution. Anti-MAP2 specifically labels neuronal cells while anti-GFAP specifically labels non-neuronal cells (e.g. glial cells). Cultures selected for transfection included << 1% of glial cells (data not shown).

Zebrafish embryo microinjection, histological analysis, and germline transmission screening

We microinjected each construct into ~200 embryos at the 1- to 2- cell stage together with Tol2 transposase RNA. The resulting EGFP reporter expression was analyzed at 24 hpf, 48 hpf 72 hpf and 96 hpf. Embryos displaying reporter expression in G0 (mosaic) embryos were raised to sexual maturity crossed to wild-type (AB strain) zebrafish and subsequently screened for germline transmission in the resulting F1 progeny.

Translation assays by polysome profiling

The location of rs3837184, a DNR in the 5'-UTR of *DPYSL2,* suggests that it could have an effect on translation efficiency. To test this possibility, we transfected HEK293 cells with 2 luciferase constructs *DPYSL2*_DNR11and 13, respectively, at about 80-90% confluence. We harvested cells after 18-24 hours post-transfection. Ten minutes before harvesting, emetine (100 μg/ml; Sigma) was added to the medium to prevent the detachment of ribosomes from the mRNA during sample preparation. We then prepared cell lysate for polysome analysis and size fractionation as previously described (Arava *et al* 2003;Stefani *et al* 2004). Briefly, HEK293 cells expressing luciferase under the control of various DNR were washed twice with ice-cold PBS containing 100 μg/ml emetine. Cells were detached using scraper and collected into 800ul of polysome lysis buffer (10 mM HEPES-KOH pH 7.4, 150mM KCl, 10 mM MgCl2, 1 mM DTT, 100 μg/ml emetine, 2% NP-40, 6 U/ml RNase inhibitor and protein inhibitor). Cell nuclei and membranes were pelleted at 13000rpm at 4°C for 10 min and the resultant cytoplasmic extracts were centrifuged through 15%-50% sucrose gradient at 40,000 rpm for 2 hours in a Beckman SW41 rotor and fractionated using Brandel Density Gradient Fractionation System with an absorbance monitor at 260 nm and 12 fractions were collected.

Zebrafish whole mount in situ hybridization results

The pattern of spatial and temporal expression of *dpysl2a and dpysl2b* genes is largely overlapping, although not identical. At 24 hpf *dpysl2a* transcript is present in the telencephalic and ventral diencephalic neuronal clusters, (Fig. 4A and supplemental Fig. 6A, B) whereas *dpysl2b* gene is expressed in the ventral and dorsal diencephalon and midbrain areas (Fig, 4B and supplemental Fig. S6C, D). Both transcripts are also present in the hindbrain, anterior and posterior lateral line (ALL and PLL, white arrowheads in Fig. 4) placodes and primary Rohon-Beard neurons (Fig. 4A, B and supplemental Fig. S6A-D).

At 48 hpf as brain develops and expands, we observed more abundant expression of both genes. While *dpysl2a* telencephalic expression fades away, there is a clear induction of *dpysl2b* transcription in this region at that

stage. Both transcripts are present in the diencephalon, midbrain tectum and tegmentum regions, as well as in the all hindbrain rhombomeres. Both genes are also expressed in the retinal ganglion cell layer. The *dpysl2a* and *dpysl2b* genes remain expressed in ALL and PLL ganglia and diffuse expression of both transcripts is observed in the spinal cord (Fig. 4G, H, M, N and supplemental Fig. S6E-H).

At 72 and 96hpf, expression becomes weaker and is confined to the anterior CNS (supplemental Fig. S6I-P). Both transcripts are rather ubiquitously expressed in all brain regions, including ganglion and internal nuclear retinal layers and cranial ganglia (supplemental Fig. S6I-P).

Fig. S1

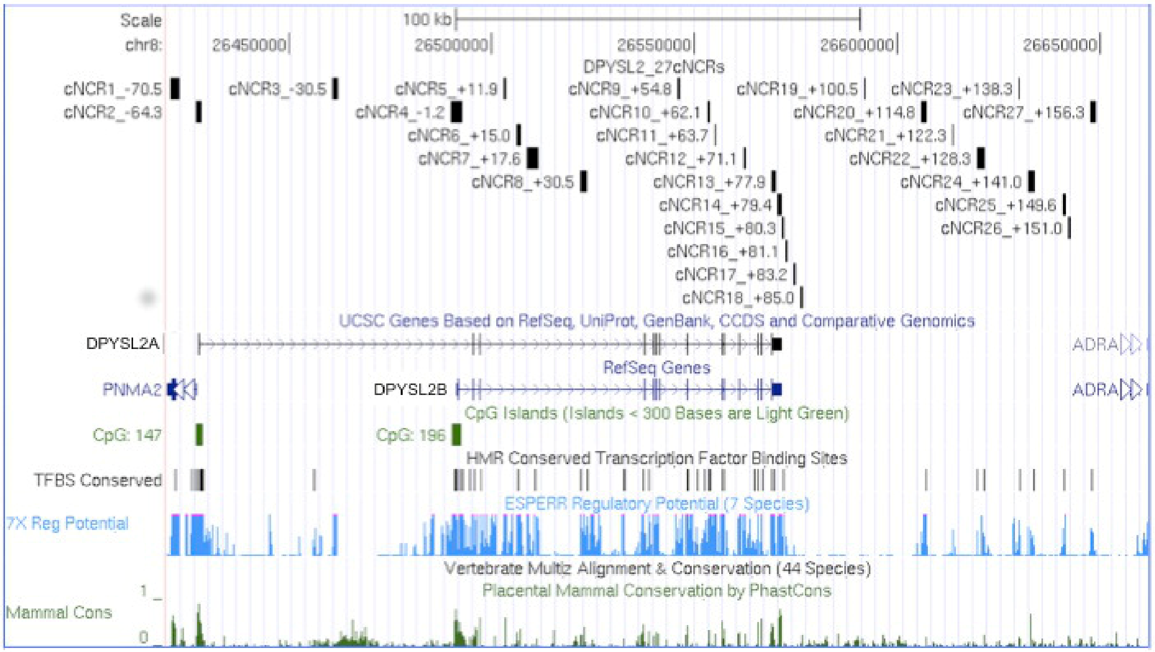

Figure S1 27 cNCRs in and around *DPYSL2* shown as custom track (black bars) on UCSC genome browser, which covered virtually all the conserved regions in an ~260kb interval in and around *DPYSL2*

Fig.S2

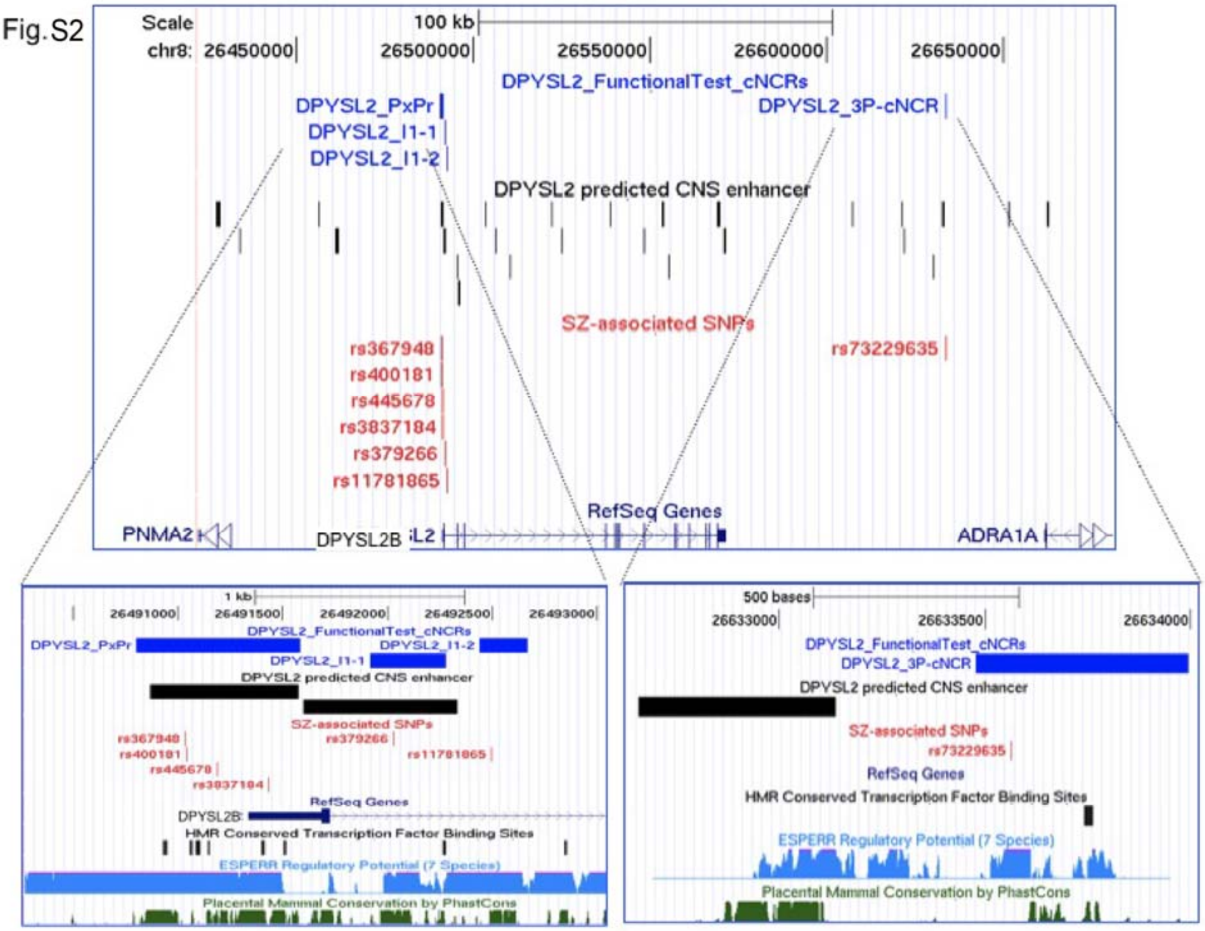

Figure S2 Four cNCRs selected for functional tests shown on UCSC genome browser in an ~260kb interval around *DPYSL2*. Blue custom track showed 4 regions (cNCRs). Black custom track showed bioinformatically predicted CNS enhancer. Red custom track showed some of those SZ-associated SNPs identified by sequencing and genotyping in the present study, and which were contained in 4 cNCRs shown in blue bars for functional tests

Fig. S3

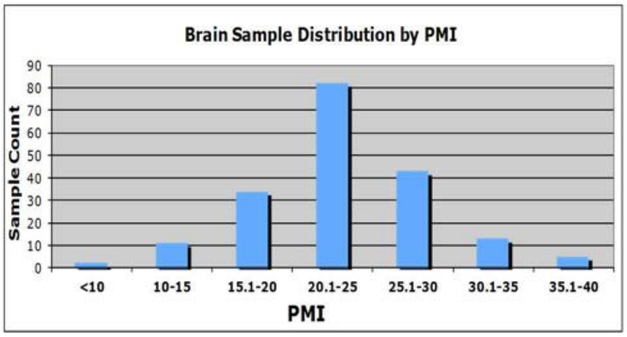

Figure S3 Distribution of PMI from 190 brain samples

Fig. S4

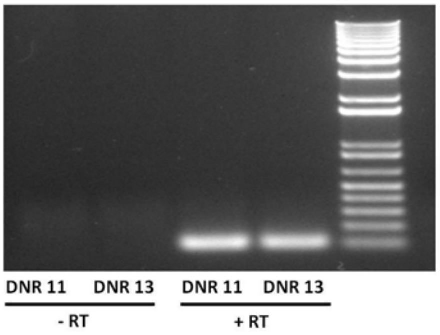

Figure S4 Negative control of RT-PCR experiment with and without reverse transcriptase when amplifying luciferase from transfected cells.

Fig.S5

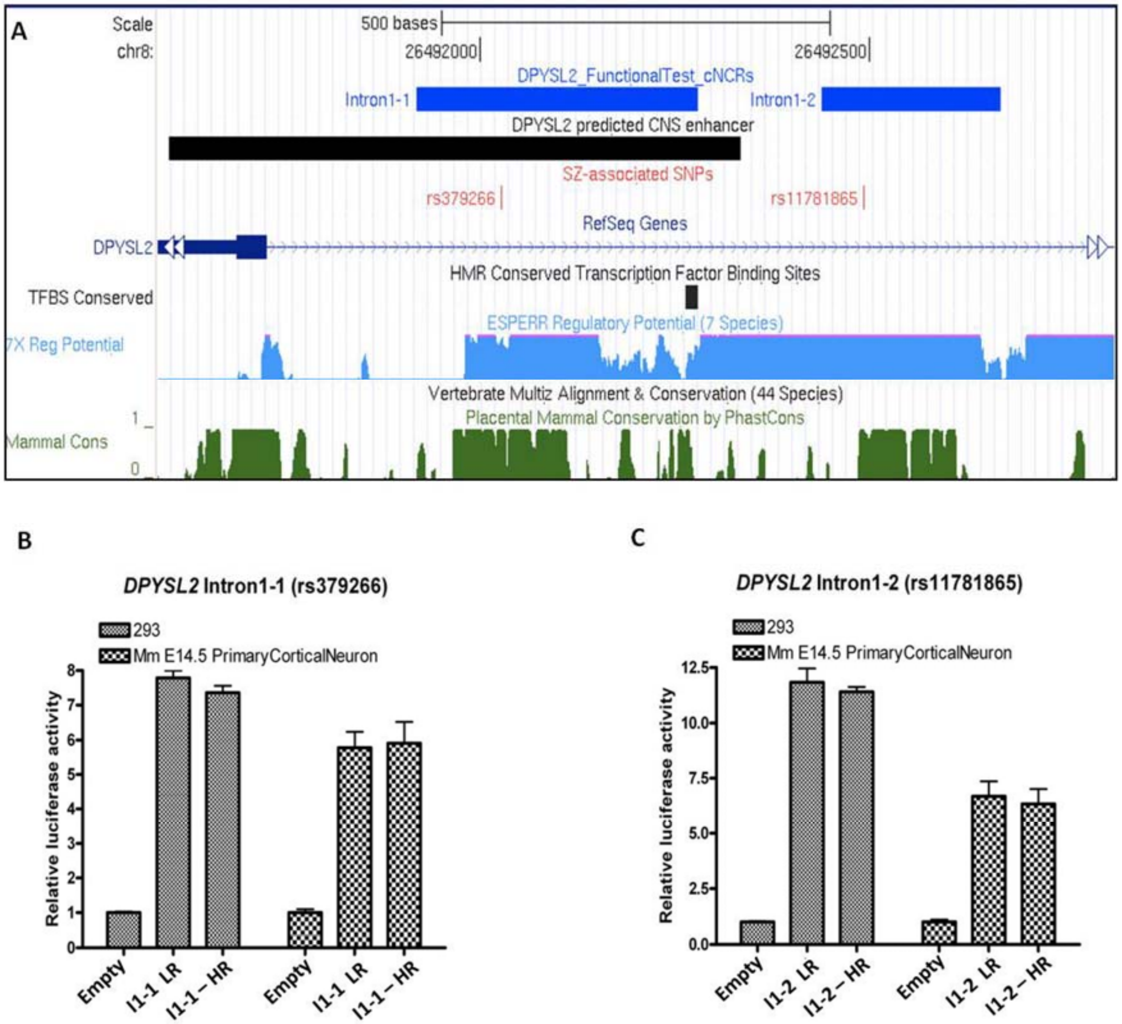

Figure S5 Human *DPYSL2* intron1 two constructs containing 2 SZ-associated SNPs did not show difference in driving luciferase expression between constructs containing Wt allele and Risk allele: A) Two constructs (blue bar) containing 2 SNPs in intron1 (red thin line) shown on the UCSC genome browser; B) *DPYSL2*_I1-1 luciferase assay results in cells; C) *DPYSL2*_I1-2 luciferase results in cells

Fig. S6

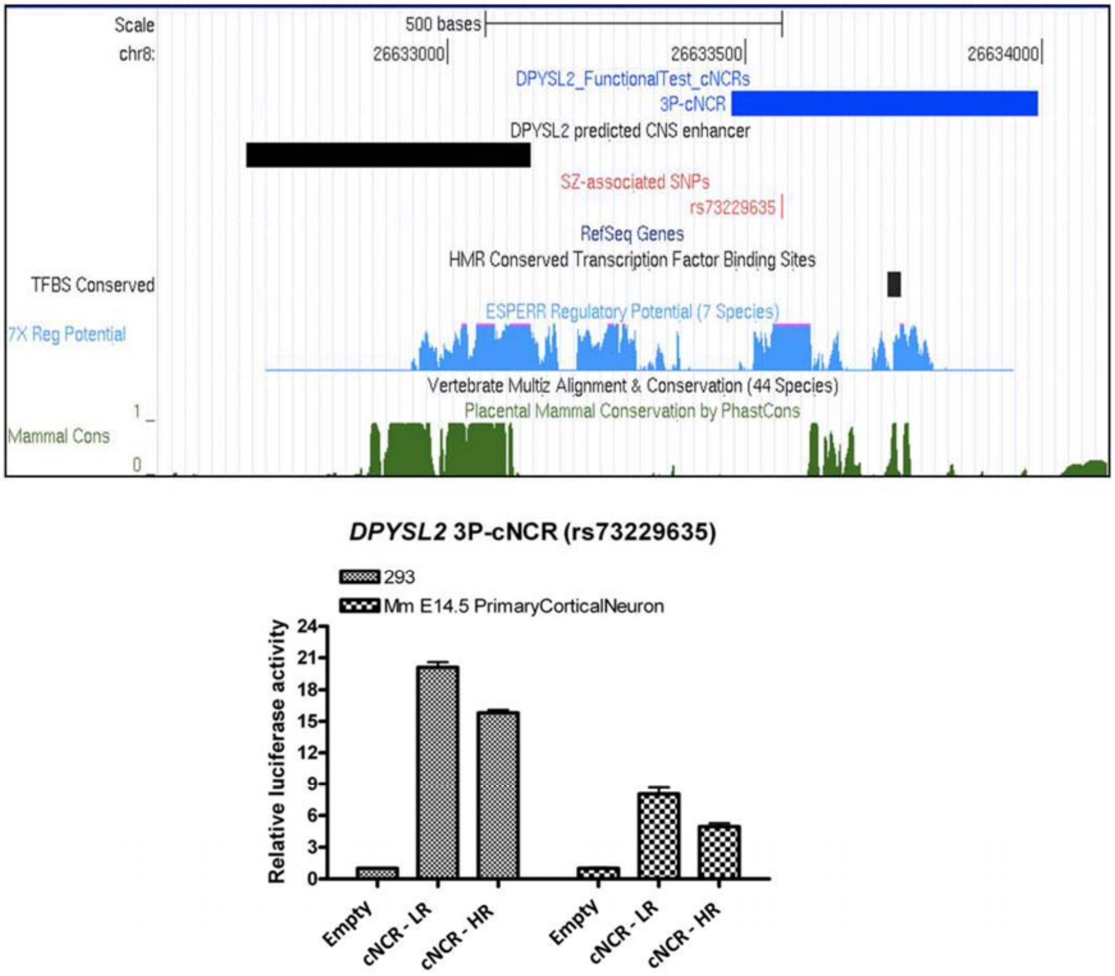

Figure S6 Human *DPYSL2* 3'-flanking cNCR (3P-cNCR) construct containing a SZ-associated SNP rs73229635 (p-value<10^{-5} in large AJ collection) showed enhancer function and difference in driving luciferase expression between constructs containing Wt allele and Risk allele: A) A construct (blue bar) containing SNP rs73229635 in 3'-flanking (red thin line) shown on the UCSC genome browser; B) 3P-cNCR luciferase assay results in cells

Fig.S7

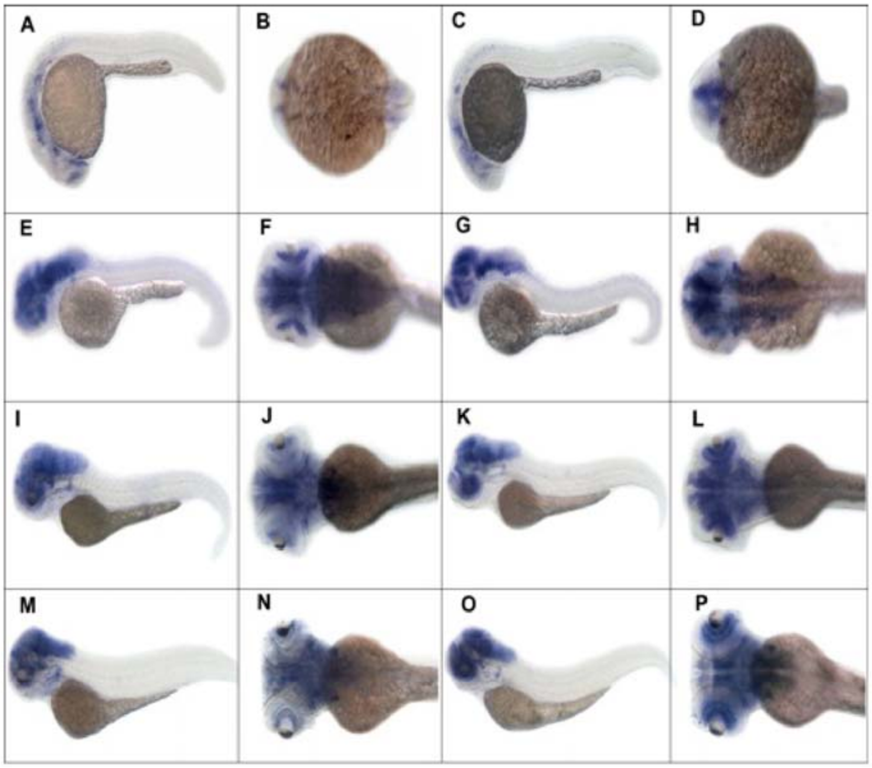

Figure S7 Whole mount *in situ* hybridization with *dpysl2a* and *dpysl2b* riboprobes. A, B, E, F, I, J, M, N – *dpysl2a in situ*; C, D, G, H, K, L, O, P – *dpysl2b in situ;* A-D – 24 hpf; E-H – 48 hpf; I-L – 72 hpf; M-P – 96 hpf; anterior is to the left; 1st and 3rd column – lateral view; 2nd and 4th column – dorsal view

Fig. S8

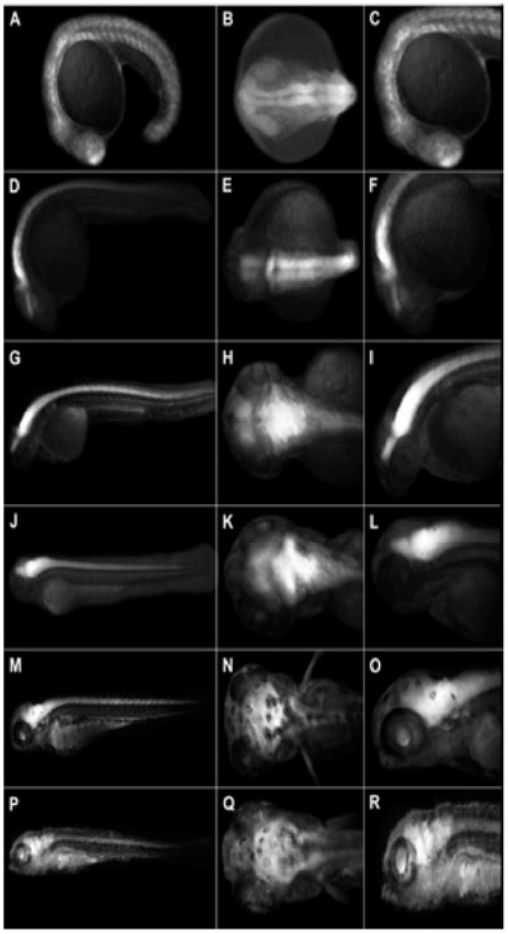

Figure S8 Transgenic zebrafish *DPYSL2*_PxPr line with *EGFP* reporter. A-C – 20 hpf; D-E – 24 hpf; G-I – 48 hpf; J-L – 72 hpf; M-O – 96hpf; P-R – 120 hpf; anterior is to the left; 1st and 3rd column – lateral view; 2nd column – dorsal view; 3rd column – lateral head magnification picture

Fig. S9

```
Mouse      ATTGCTGTAGTTTATTTCCACCCCCTTCCCTCCTGTT---------------------CT
Rat        ATTGCTGTAGTTTATTTCCACCCCCTTCCCTCCTGTT---------------------CT
Human      ATTGCTGTAGTTTATTTCCACCCCCTTCCCTCCTGTTTCTCTCTCCTTCTCTCTCTCT
           *************************************                     **

Mouse      CTCTCCCTCT-T-TCTTTTTTCCGCCCTAGCTGGATCTGTGTTGGAGGAGAG----GAAA
Rat        CTCTCCCTCTCTTTCTTTTTTCCGCCCTAGCTGGATCTGTGTTGGAGGAGAG----AAAA
Human      CTCTCTCTCTCTCTTTTTTTTCCGCCCTAGCTGGGGCTGTGTTGGAGGAGAGGAAGAAAG
           ***** **** * * *******************  ****************     **

Mouse      AGAGACAGAGGATTGCATCTG----------TTTTGAAATCTCCTAATAGCAAGACCAGT
Rat        AGAGACAGAGGATTGCATCTGTCTCTTATAGTTTGAAATCTCCTAATAGCAAGACCAGC
Human      AGAGACAGAGGATTGCATTCATCCGTTACGTTCTTGAAATTTCCTAATAGCAAGACCAGC
           ******************             * ******* ******************

Mouse      TAAGGGATTGTACCTTTTTCCTAC-----------AAATATAAATATATATATATTTTAAA
Rat        TAAGGGATTGTACCTTTTTCCTAC-----------AAATATAAATATATATATATTTTAAA
Human      GAAGCGGTTGCACCCTTTTCAATCTTGCAAAGGAAAAAAACAAAACAAAACAAAAAAAAC
            *** * *** *** *****   *          *** *  ** * * *   *    **

Mouse      CCAAGTCTTTTTTTTTCCGGCTATCTTTGCTTTAAAGCTGTCCTCTTGAGATTACTTCCT
Rat        CCAAGTCTTTTTTT--CCGGCTCTCTTTGCTTTAAAGCTGTCCTCTTGAAATTACTTCCC
Human      CCAAGTCCCCTTCC---CGGCAGTTTTGCCTTAAAGCTGCCCTCTTGAAATTAATTTTT
           *******   **     ****  * ***** ********* ******** **** **

Mouse      CCCGCCCCCCGGAGAGATG
Rat        CCCGCCCCCCGGAGAGATG
Human      TCC---CAGGAGAGAGATG
            **        ********
```

Figure S9 Sequences of human and rat *DPYSL2* showing 5'-TOP sequence and polymorphic DNR in human sequence

Table S1 Sample counts for sequencing and genotyping

	SZ Independent cases		Independent Controls		
	CEU	AJ	CEPH	AJ	
				EpiGen	NYCP
Sequencing	48	89	96	55	
Genotyping		729		821	721

Table S2 Primers for *DPYSL2* 14 exons and 27 cNCRs

Primer_ID	Primer-Seq	Genomic location	Product(bp)
DPYSL2 E1 F	AATCGCTGCTCGTCTCTCTC	chr8:26491276-26491829	554
DPYSL2 E1 R	AGTTCGGTCCCTCTCCTTTC		
DPYSL2 E2 F	GGTTGCCACATACAGGCTTA	chr8:26495239-26495602	364
DPYSL2 E2 R	TGGTCACTCTCTGATGTTCCTG		
DPYSL2 E3 F	GCACAGAGCGGAGGATAGTT	chr8:26497129-26497521	393
DPYSL2 E3 R	GTGGCTCAGGACAGCAAGTT		
DPYSL2 E4 F	CGATTTTGAACCCAAGAAGC	chr8:26537468-26537843	376
DPYSL2 E4 R	ACAAATGGGATCTGGAAGCA		
DPYSL2 E5-7-1 F1	TGCTTCCTTTACCATTCTGTG	chr8:26539859-26540409	551
DPYSL2 E5-7-1 R1	AAGCTCTTGGGTGACACCTG		
DPYSL2 E5-7-2 F	CGGCACTCAGAACCTCCTTA	chr8:26540352-26540844	493
DPYSL2 E5-7-2 R	ACCAGGTCCCTCTGCTTTTC		
DPYSL2 E5-7-3 F	CAGGGCTTTCTTTTTCGTCA	chr8:26540732-26541264	533
DPYSL2 E5-7-3 R	ATTTTGCACAGCTCTGATGG		
DPYSL2 E5-7-4 F	GAACGATCGGCAGTGGTAAT	chr8:26541186-26541597	412
DPYSL2 E5-7-4 R	ATCTTCTGGCGAAACAGTCC		
DPYSL2 E8 F	CAGCCTCGCCTTCATCTTAG	chr8:26548111-26548535	425
DPYSL2 E8 R	ATACTAACAAGGCCGCAGCA		
DPYSL2 E9 F	TCTGCGAGATGAGCCTGATA	chr8:26556732-26557152	421
DPYSL2 E9 R	TTTGGTGAACAAGGGTCTCC		
DPYSL2 E10 F	GGGAGGGGATTCTGGATAAA	chr8:26557201-26557618	418
DPYSL2 E10 R	TGTCATTTCAAGGCCTCCAT		
DPYSL2 E11 F	CAACGCTCTTGACATCCATC	chr8:26560945-26561380	436
DPYSL2 E11 R	CCCAGCAAATTGCAAATCAT		
DPYSL2 E12 F	CAGGATCCCTGTCTCTGAGTCT	chr8:26565570-26565997	428
DPYSL2 E12 R	GCTCCCTCTTATCCCTGGAA		
DPYSL2 E13 F	CACACAACACCTGTCCACCT	chr8:26566525-26566956	432
DPYSL2 E13 R	CACGTTGGAGAGAAAGGGAAT		
DPYSL2 E14 F	GCACTTTTCCTCCTGAGCTG	chr8:26568950-26569384	435
DPYSL2 E14 R	GGTGGGTAGTCTTGGGTGTG		

cNCR1_-70.5_F	CTGCAGTGAGCCGAGATTTT	chr8:26,420,806-26,423,180	2375
cNCR1_-70.5_R	TGCATTAACGGTCTTCCTACTG		
cNCR2_-64.4_F	AAGGCGAGTGGACAAACTGA	chr8:26,426,916-26,428,447	1532
cNCR2_-64.4_R	CGGGAGGGTTTCAGAAGAAT		
cNCR3_-30.5_F	TACTGGAGCAGAAGCCCACT	chr8:26,460,813-26,462,243	1431
cNCR3_-30.5_R	GACCACCAGGCTCAAGTGAT		
cNCR4_-1.2_F	CTGGGCAGTCAGGACACTCT	chr8:26,490,109-26,492,741	2633
cNCR4_-1.2_R	GAGAGGTGGGAGCAAGACTG		
cNCR5_+11.9_F	AAATCACATGGTCCCAGCAC	chr8:26,503,272-26,503,801	530
cNCR5_+11.9_R	CCCGTGTCCTTAGGAGAAATG		
cNCR6_+15.0_F	CCCCAAGCTGACCTTTCTCT	chr8:26,506,341-26,507,564	1223
cNCR6_+15.0_R	CACGCTCAGACAGAAAGCAC		
cNCR7_+17.6_F	GCATTCAAATGAGGCCAACT	chr8:26,508,894-26,511,818	2925
cNCR7_+17.6_R	TCAGGAGATGGAGAGGAGGA		
cNCR8_+30.5_F	GAGGGATACTTTGCATCTGGA	chr8:26,521,874-26,52,3744	1871
cNCR8_+30.5_R	TCCCAACAGAATGTATCAGGAA		
cNCR9_+54.8_F	CTGTATCTCTGGGAGGTGCTG	chr8:26,546,129-26,546,478	350
cNCR9_+54.8_R	CCTTGTCTCCACCAGAGCTT		
cNCR10_+62.1_F	CTGTCCATCCCAGTTGGTTT	chr8:26,553,478-26,554,076	599
cNCR10_+62.1_R	CAGGCCTCTACCTCCACAAG		
cNCR11_+63.7_F	GATGAACCGGAGTGGTTTGT	chr8:26,555,009-26,555,563	555
cNCR11_+63.7_R	TCCAAGCTGGAATTTGATCC		
cNCR12_+71.1_F	GGGTAGGTTGGGGTGAAAAT	chr8:26,562,422-26,562,930	509
cNCR12_+71.1_R	TGACTTCTTGGGTGTCCTGA		
cNCR13_+77.9_F	TACTGTGGAGCAGCCAGTTC	chr8:26,569,258-26,570,280	1023
cNCR13_+77.9_R	GGTTCTGCAGCCTCATACCT		
cNCR14_+79.4_F	TCCATCCTTCCCTCTCCTTT	chr8:26,570,707-26,571,758	1052
cNCR14_+79.4_R	TAGCAGCCTGTGACATGGAG		
cNCR15_+80.3_F	TGTGGTTGTTTCAGGGGTGT	chr8:26,571,596-26,572,220	625
cNCR15_+80.3_R	CCCAAGGCACACACTCTCTT		
cNCR16_+81.1_F	ATATTGCCTGGGCTGGTTTT	chr8:26,572,480-26,573,107	628
cNCR16_+81.1_R	CCCATCTTGGTGTGAGATGA		

cNCR17_+83.2_F	AGCCAAGATCGTGCCACTTA	chr8:26,574,557-26,575,013	457
cNCR17_+83.2_R	CCTGTGTGGTGCAGAGATGT		
cNCR18_+85.0_F	TGGTAATGGCCGCTTAAGTT	chr8:26,576,327-26,576,849	523
cNCR18_+85.0_R	TACAGGCTGTCCCTGTCCTT		
cNCR19_+100.5_F	GAGGGTCTCCTTCCTGCTCT	chr8:26,591,864-26,592,384	521
cNCR19_+100.5_R	CTGGAATTGGGAGGGAAGTT		
cNCR20_+114.8_F	AGATCAGGCCTCCTCTACCC	chr8:26,606,089-26,607,484	1396
cNCR20_+114.8_R	CAGCGATATGCAAAATGAGG		
cNCR21_+122.3_F	CTCCCGAGTTGCTTGTGAAT	chr8:26,613,669-26,614,069	401
cNCR21_+122.3_R	TCAGGACTTTCAAGGCTGCT		
cNCR22_+128.3_F	CTGCACCCAGTGATGTGTGT	chr8:26,619,609-26,621,844	2236
cNCR22_+128.3_R	TGCTGTTGTCCTCTGTGAAAA		
cNCR23_+138.3_F	AAGCATTCTCTGTGGGAGTCA	chr8:26,630,003-26,630,401	399
cNCR23_+138.3_R	TTCCCATCTTTTGCAGTAGGA		
cNCR24_+141.0_F	GAGTTTGCCTCTGGGAAGTG	chr8:26,632,377-26,633,994	1618
cNCR24_+141.0_R	CTGGCTTTTGATCTCGGTTC		
cNCR25_+149.6_F	CCTTCCTGCCAATTCTCTGA	chr8:26,640,955-26,641,598	644
cNCR25_+149.6_R	TCCCAAAGTGCTGGGATTAG		
cNCR26_+151.0_F	CCCCCACACCTTTCTTTCTT	chr8:26,642,317-26,642,906	590
cNCR26_+151.0_R	TACTAATACAAGCCTTATACCCTGTGC		
cNCR27_+156.3_F	GCACCCTATTCTCTCTCCTCCT	chr8:26,647,655-26,649,235	1581
cNCR27_+156.3_R	GGCCTTTGAAGGAAACCTCT		

Table S3 Four cNCRs selected for functional tests

cNCR Name<br>Genomic coordinates	Contruct name	SNPs covered	Allele(s)of SNPs	Length (bp)
***DPYSL2*_PxPr**<br>26490803-26491584	DPYSL2_Pr3SNP_LR<br>DPYSL2_Pr3SNP_HR	rs367948, rs400181<br>rs445678, rs3837184	CTT+11TC<br>GCC+11TC	782
	DPYSL2_5'DNR_LR	rs367948, rs400181<br>rs445678, rs3837184	CTT+11TC<br>GCC+11TC	782
	DPYSL2_5'DNR_HR	rs367948, rs400181<br>rs445678, rs3837184	CTT+12TC<br>GCC+12TC<br>CTT+13TC<br>GCC+13TC<br>CTT+14TC<br>GCC+14TC	
***DPYSL2*_I1-1**<br>26491918-26492279	DPYSL2_I1-1_LR<br>DPYSL2_I1-1_HR	rs379266	T<br>C	362
***DPYSL2*_I1-2**<br>26492439-26492669	DPYSL2_I1-2_LR<br>DPYSL2_I1-2_HR	rs11781865	G<br>C	231
***DPYSL2*_3P-cNCR**<br>26633478-26633994	DPYSL2_3P-cNCR_LR<br>DPYSL2_3P-cNCR_HR	rs73229635	T<br>C	517

note: LR stands for low risk; HR stands for high risk

Table S4 Brain samples

Distribution by sex and plates

SEX	Plate1	Plate2	Total
Female	22	21	43
Male	75	72	147
Total	97	93	190

Distribution by age and plates

AGE	Plate1	Plate2	Total
<20	2	0	2
21-30	2	2	4
31-40	1	2	3
41-50	5	5	10
51-60	31	28	59
61-70	28	29	57
71-80	24	24	48
>80	4	3	7
Total	97	93	190

Table S5 Primers used for real time PCR

Primer Name	Primer sequence	Length(bp)	Genome coordinates (hg18)
Human_DPYSL2B_F	AAAATATTCCACGCATCACG	113bp (DPYSL2B_F/R)	chr8:26491707-26491726
Human_DPYSL2A_F	AAGCCCTGCAGAACATCAAC	119bp (DPYSL2A_F/R)	chr8:26428087-26428106
Human_DPYSL2_R	TTGCTTGATCAACCCATCTTC		chr8:26495474-26495494

Table S6 The list of 120 variants in and around *DPYSL2* identified by sequencing

Variant	Variant ID	Sequence context	genomic coordinate (dbSNP129)	genomic coordinate (dbSNP135)
1	rs2233699	5' end cNCR	26422670	26366753
2	rs17403251	5' end cNCR	26427460	26371543
3	rs149517015	5' end cNCR	26428268	26372351
4	rs9314324	5' end cNCR	26460877	26404960
5	rs13279683	5' end cNCR	26460941	26405024
6	rs78025765	5' end cNCR	26461102	26405185
7	rs9314325	5' end cNCR	26461170	26405253
8	rs7831864	5' end cNCR	26461425	26405508
9	rs12548421	5' end cNCR	26461547	26405630
10	rs12543392	5' end cNCR	26461556	26405639
11	rs12543398	5' end cNCR	26461608	26405691
12	rs10110479	5' end cNCR	26461939	26406022
13	rs431246	5' end cNCR(PxPr)	26490712	26434795
14	rs367948	5' end cNCR(PxPr)	26491033	26435116
15	rs400181	5' end cNCR(PxPr)	26491040	26435123
16	rs445678	5' end cNCR(PxPr)	26491188	26435271
17	rs3837184	5'-UTR DNR	26491428	26435511
18	rs58966940	5'-UTR	26491458	26435541
19	rs379266	intronic cNCR	26492026	26436109
20	rs11285674	intronic cNCR	26492389	26436472
21	rs11781865	intronic cNCR	26492493	26436576
22	rs2584184	intronic cNCR	26492687	26436770
23	rs415524	intronic cNCR	26497192	26441275
24	rs11786691	coding synonymous	26497394	26441477
25	rs408753	intronic cNCR	26497479	26441562
26	rs17055482	intronic cNCR	26503398	26447481
27	rs140053560	intronic cNCR	26503656	26447739
28	rs62491913	intronic cNCR	26503707	26447790
29	rs68019818	intronic cNCR	26503747	26447830
30	rs7825468	intronic cNCR	26506390	26450473
31	rs146056239	intronic cNCR	26506775	26450858
32	rs5029306	intronic cNCR	26509022	26453105
33	rs7820433	intronic cNCR	26521976	26466059
34	rs113199330	coding synonymous	26537583	26481666
35	rs327222	coding synonymous	26537688	26481771
36	rs139123217	coding synonymous	26537739	26481822
37	rs2289592	intronic cNCR	26540291	26484374
38	un26540309	intronic cNCR	26540309	26484392
39	un26540321	intronic cNCR	26540321	26484404
40	rs17322275	intronic cNCR	26540530	26484613
41	rs327228	intronic cNCR	26540979	26485062
42	rs73678824	intronic cNCR	26540980	26485063
43	R227R	coding synonymous	26541364	26485447
44	rs753508	intronic cNCR	26546168	26490251

45	rs327217	intronic cNCR	26546233	26490316
46	rs35621323	coding synonymous	26548248	26492331
47	rs78121726	coding synonymous	26548314	26492397
48	rs148064770	coding nonsynonymous	26548321	26492404
49	rs55906521	intronic cNCR	26548387	26492470
50	rs1867042	intronic cNCR	26548477	26492560
51	rs77434938	intronic cNCR	26553737	26497820
52	rs13277175	intronic cNCR	26553930	26498013
53	rs327218	intronic cNCR	26555144	26499227
54	rs118022714	intronic cNCR	26555248	26499331
55	rs62493399	intronic cNCR	26555414	26499497
56	rs139163162	coding synonymous	26561224	26505307
57	rs11776801	intronic cNCR	26561368	26505451
58	rs9644117	intronic cNCR	26561435	26505518
59	rs708621	coding synonymous	26566709	26510792
60	rs147699216	coding synonymous	26569064	26513147
61	un26569149	3'-UTR cNCR	26569149	26513232
62	rs58827210	3'-UTR cNCR	26570018	26514101
63	rs17055639	3'-UTR cNCR	26570061	26514144
64	rs17055641	3'-UTR cNCR	26570088	26514171
65	rs1058332	3'-UTR cNCR	26570210	26514293
66	rs11863	3'-UTR cNCR	26570233	26514316
67	rs45471201	3'-UTR cNCR	26571055	26515138
68	rs17666	3'-UTR cNCR	26571375	26515458
69	rs10495	3'-UTR cNCR	26571389	26515472
70	rs10042	3'-UTR cNCR	26571580	26515663
71	rs7827731	3'-end cNCR	26571810	26515893
72	rs7828056	3'-end cNCR	26571955	26516038
73	rs7831883	3'-end cNCR	26572130	26516213
74	rs57045236	3'-end cNCR	26572888	26516971
75	rs7845740	3'-end cNCR	26574668	26518751
76	rs7842128	3'-end cNCR	26574697	26518780
77	rs58047393	3'-end cNCR	26574771	26518854
78	rs57846118	3'-end cNCR	26574826	26518909
79	rs56322416	3'-end cNCR	26574827	26518910
80	rs12155555	3'-end cNCR	26576477	26520560
81	rs6557935	3'-end cNCR	26576551	26520634
82	rs186111033	3'-end cNCR	26592043	26536126
83	un26592058	3'-end cNCR	26592058	26536141
84	rs7831201	3'-end cNCR	26592060	26536143
85	rs187288868	3'-end cNCR	26606195	26550278
86	rs112917653	3'-end cNCR	26606207	26550290
87	rs73678614	3'-end cNCR	26606441	26550524
88	un26606675	3'-end cNCR	26606675	26550758
89	un26606723	3'-end cNCR	26606723	26550806
90	rs17330796	3'-end cNCR	26607314	26551397
91	rs17055703	3'-end cNCR	26607333	26551416
92	un26613860	3'-end cNCR	26613860	26557943
93	rs17422356	3'-end cNCR	26614030	26558113

94	rs115507492	3'-end cNCR	26621427	26565510
95	rs191918596	3'-end cNCR	26621583	26565666
96	rs7007717	3'-end cNCR	26621603	26565686
97	rs7001655	3'-end cNCR	26621687	26565770
98	rs34790461	3'-end cNCR	26621771	26565854
99	rs13278281	3'-end cNCR	26632938	26577021
100	rs73229635	3'-end cNCR	26633562	26577645
101	rs114104363	3'-end cNCR	26633800	26577883
102	rs994098	3'-end cNCR	26633836	26577919
103	rs1036813	3'-end cNCR	26633934	26578017
104	rs6998714	3'-end cNCR	26641066	26585149
105	rs4732831	3'-end cNCR	26641094	26585177
106	un26641095	3'-end cNCR	26641095	26585178
107	rs10503796	3'-end cNCR	26641185	26585268
108	rs1908650	3'-end cNCR	26641530	26585613
109	rs1025859	3'-end cNCR	26647800	26591883
110	rs78167668	3'-end cNCR	26648009	26592092
111	rs1025858	3'-end cNCR	26648036	26592119
112	rs2132449	3'-end cNCR	26648270	26592353
113	rs1016733	3'-end cNCR	26648327	26592410
114	rs1016734	3'-end cNCR	26648421	26592504
115	rs76802184	3'-end cNCR	26648455	26592538
116	rs35551735	3'-end cNCR	26648560	26592643
117	rs17055838	3'-end cNCR	26649076	26593159
118	rs111576315	3'-end cNCR	26649096	26593179
119	rs191064950	3'-end cNCR	26649117	26593200
120	un26649188	3'-end cNCR	26649188	26593271

Note: novel variants are named after their genomic coordinates in dbSNP129
