# Supplementary material for: Functional Variants in DPYSL2 Sequence Increase Risk of Schizophrenia and Suggest a Link to mTOR Signaling
Source: G3 (Bethesda). 2014 Nov 20;5(1):61–72. doi: 10.1534/g3.114.015636 (PMC4291470; doi:10.1534/g3.114.015636)
Supplement: Supporting Information [file supp_g3.114.015636_FileS1.pdf]

File S1

SUPPLEMENTAL MATERIALS

Study subjects

Our CEU multiplex families were recruited from multiple sources including our Maryland Epidemiologic Samples (MES) (Pulver and Bale 1989), our nationwide advertising efforts, and national and international collaborators (US, Italy, Poland, Greece). All families are of European-Caucasian ancestry. Descriptions of our clinical assessment methods are published elsewhere (Blouin *et al* 1998).

Our AJ SZ and controls were recruited in North America by advertisements in newspapers and Jewish newsletters, talks to community organizations, letters to leaders of the Jewish community, letters and talks to service providers and a study website hosted by the Johns Hopkins Epidemiology-Genetics Program (EpiGen) in Psychiatry (Fallin *et al* 2003). AJ SZ cases and controls self-identified Ashkenazi Jewish ethnicity in all four grandparents.

All CEU and AJ SZ cases met probable or definite DSM-IV schizophrenia or schizoaffective disorder criteria based on a consensus diagnosis. Our diagnostic methods are available in detail (Fallin *et al* 2005). All controls collected by the MES screened negative for a history of psychosis, mania, psychiatric hospitalization, depression or suicide attempts. All recruitment methods and protocols for collection of clinical data and blood samples were approved by the Johns Hopkins Institutional Review Board; informed consent was obtained from all subjects.

In the present study, we used an additional 721 controls of AJ descent from New York Cancer Project (NYCP) Biorepository (Foulkes *et al* 2002); the ethnicity of their grandparents is less certain.

Sequencing subjects

We selected 48 CEU SZ probands who were homozygous for the risk alleles for the SNP with high linkage signal. We sequenced 96 unrelated Centre d'Etude du Polymorphisme Humain (CEPH) samples as a CEU control. We chose 48 AJ SZ subjects from our sample because they have a positive family history for psychosis: i.e., at least two members of the family (the proband and one more first and second degree relatives) had evidence of psychosis either from direct assessment or from family informant data. The 48 AJ controls were all 40 year of age or greater (an age at which the risk of developing SZ is reduced by 90%) with no history of depression, mania, psychosis, or psychiatric hospitalization. We selected an additional 41 AJ SZ probands who were homozygous for the risk alleles at

both peak associated SNPs (rs12155555; rs5029306) and 7 AJ controls who were homozygous for the non-risk alleles at both rs12155555 and rs5029306 to identify variants segregating on the risk haplotype and to detect variants that might be specific to the 41 AJ SZ probands.

SNP selection and genotyping

The selected 10 SNPs are rs445678 in the promoter region, rs408753 in intron 3, un26537739, a novel synonymous (H107H), rs55906521 in intron 8, un26569149, a novel noncoding variant, 10bp from stop codon, rs10042, rs17055641, rs45471201 in the 3'-UTR, rs57045236, an 11bp insertion/deletion, ~1kb from 3' of *DPYSL2*, and finally rs73229635, a noncoding variant, ~62kb from 3' of *DPYSL2*.

HEK 293 cells culture

We cultured HEK293 cells in Dulbecco's modified Eagle's medium (DMEM) supplemented with 10% fetal bovine serum (FBS) and 1% penicillin and streptomycin in a humidified 5% CO_2 atmosphere at 37°C.

Primary embryonic day 14.5 (E14.5) mouse cortical neuronal cell culture

We dissociated cells from E14.5 mouse cerebral cortices and seeded cells on poly-D-lysine-coated 24-well plates in plating medium (Opti-MEM containing 10% horse serum, 1% glutamax I, 1% penicillin-streptomycin, 1% N2 supplement, and 2.5M glucose). After 3 hours, we changed to maintenance medium (neurobasal medium containing 1% glutamax I, 2% B27 supplement, and 1% penicillin-streptomycin) and replaced half of maintenance medium with fresh maintenance medium every other day.

Immunofluorescence staining

On the 3rd day of culture, we characterized the E14.5 mouse cortical neurons by immunostaining. Cells were fixed in 4% paraformaldehyde and permeabilized in 0.3% Triton and then incubated with anti-MAP2 (chicken polyclonal antibody, Abcam) and anti-GFAP (rabbit polyclonal antibody, Fisher Scientific) diluted at 1:100, followed by incubation with fluorescently labeled Goat anti-chicken/rabbit secondary antibodies (Molecular Probe) at a 1:500 dilution. Anti-MAP2 specifically labels neuronal cells while anti-GFAP specifically labels non-neuronal cells (e.g. glial cells). Cultures selected for transfection included << 1% of glial cells (data not shown).

Zebrafish embryo microinjection, histological analysis, and germline transmission screening

We microinjected each construct into ~200 embryos at the 1- to 2- cell stage together with Tol2 transposase RNA. The resulting EGFP reporter expression was analyzed at 24 hpf, 48 hpf 72 hpf and 96 hpf. Embryos displaying reporter expression in G0 (mosaic) embryos were raised to sexual maturity crossed to wild-type (AB strain) zebrafish and subsequently screened for germline transmission in the resulting F1 progeny.

Translation assays by polysome profiling

The location of rs3837184, a DNR in the 5'-UTR of *DPYSL2,* suggests that it could have an effect on translation efficiency. To test this possibility, we transfected HEK293 cells with 2 luciferase constructs *DPYSL2*_DNR11and 13, respectively, at about 80-90% confluence. We harvested cells after 18-24 hours post-transfection. Ten minutes before harvesting, emetine (100 µg/ml; Sigma) was added to the medium to prevent the detachment of ribosomes from the mRNA during sample preparation. We then prepared cell lysate for polysome analysis and size fractionation as previously described (Arava *et al* 2003;Stefani *et al* 2004). Briefly, HEK293 cells expressing luciferase under the control of various DNR were washed twice with ice-cold PBS containing 100 µg/ml emetine. Cells were detached using scraper and collected into 800ul of polysome lysis buffer (10 mM HEPES-KOH pH 7.4, 150mM KCl, 10 mM MgCl2, 1 mM DTT, 100 µg/ml emetine, 2% NP-40, 6 U/ml RNase inhibitor and protein inhibitor). Cell nuclei and membranes were pelleted at 13000rpm at 4°C for 10 min and the resultant cytoplasmic extracts were centrifuged through 15%-50% sucrose gradient at 40,000 rpm for 2 hours in a Beckman SW41 rotor and fractionated using Brandel Density Gradient Fractionation System with an absorbance monitor at 260 nm and 12 fractions were collected.

Zebrafish whole mount in situ hybridization results

The pattern of spatial and temporal expression of *dpysl2a and dpysl2b* genes is largely overlapping, although not identical. At 24 hpf *dpysl2a* transcript is present in the telencephalic and ventral diencephalic neuronal clusters, (Fig. 4A and supplemental Fig. 6A, B) whereas *dpysl2b* gene is expressed in the ventral and dorsal diencephalon and midbrain areas (Fig, 4B and supplemental Fig. S6C, D). Both transcripts are also present in the hindbrain, anterior and posterior lateral line (ALL and PLL, white arrowheads in Fig. 4) placodes and primary Rohon-Beard neurons (Fig. 4A, B and supplemental Fig. S6A-D).

At 48 hpf as brain develops and expands, we observed more abundant expression of both genes. While *dpysl2a* telencephalic expression fades away, there is a clear induction of *dpysl2b* transcription in this region at that

stage. Both transcripts are present in the diencephalon, midbrain tectum and tegmentum regions, as well as in the all hindbrain rhombomeres. Both genes are also expressed in the retinal ganglion cell layer. The *dpysl2a* and *dpysl2b* genes remain expressed in ALL and PLL ganglia and diffuse expression of both transcripts is observed in the spinal cord (Fig. 4G, H, M, N and supplemental Fig. S6E-H).

At 72 and 96hpf, expression becomes weaker and is confined to the anterior CNS (supplemental Fig. S6I-P). Both transcripts are rather ubiquitously expressed in all brain regions, including ganglion and internal nuclear retinal layers and cranial ganglia (supplemental Fig. S6I-P).
