# Supplementary material for: Functional Variants in DPYSL2 Sequence Increase Risk of Schizophrenia and Suggest a Link to mTOR Signaling
Source: G3 (Bethesda). 2014 Nov 20;5(1):61–72. doi: 10.1534/g3.114.015636 (PMC4291470; doi:10.1534/g3.114.015636)
Supplement: Supporting Information [file supp_g3.114.015636_FigureS1.pdf]

Fig. S1

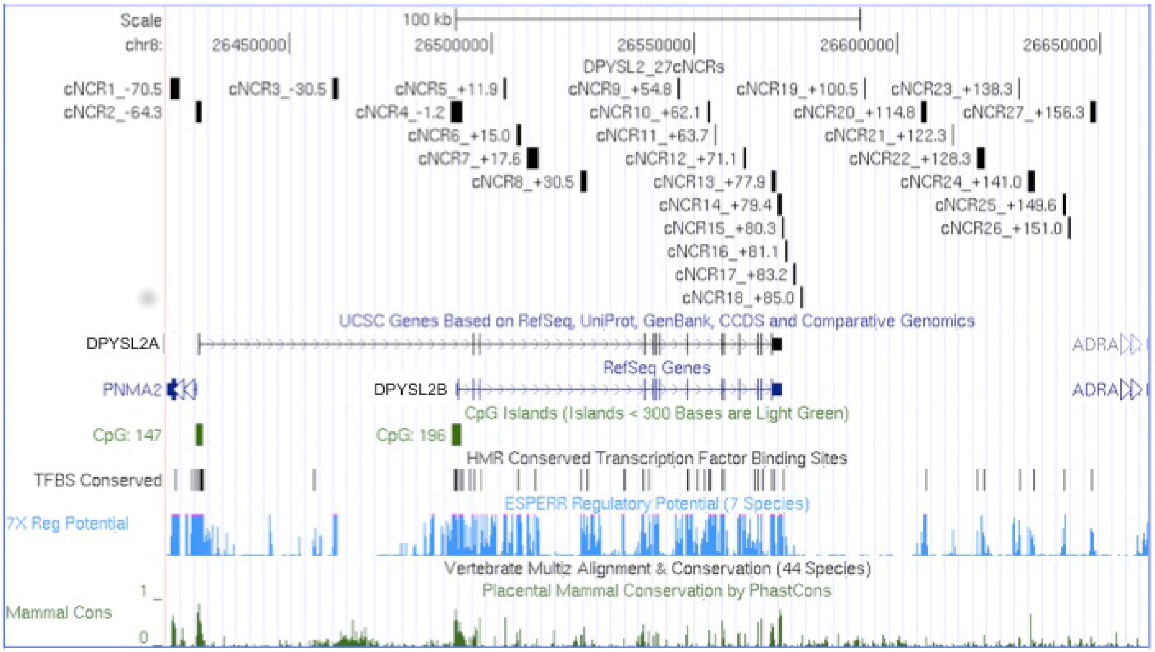

Figure S1 27 cNCRs in and around *DPYSL2* shown as custom track (black bars) on UCSC genome browser, which covered virtually all the conserved regions in an ~260kb interval in and around *DPYSL2*
