# Supplementary material for: Functional Variants in DPYSL2 Sequence Increase Risk of Schizophrenia and Suggest a Link to mTOR Signaling
Source: G3 (Bethesda). 2014 Nov 20;5(1):61–72. doi: 10.1534/g3.114.015636 (PMC4291470; doi:10.1534/g3.114.015636)
Supplement: Supporting Information [file supp_g3.114.015636_FigureS2.pdf]

Fig.S2

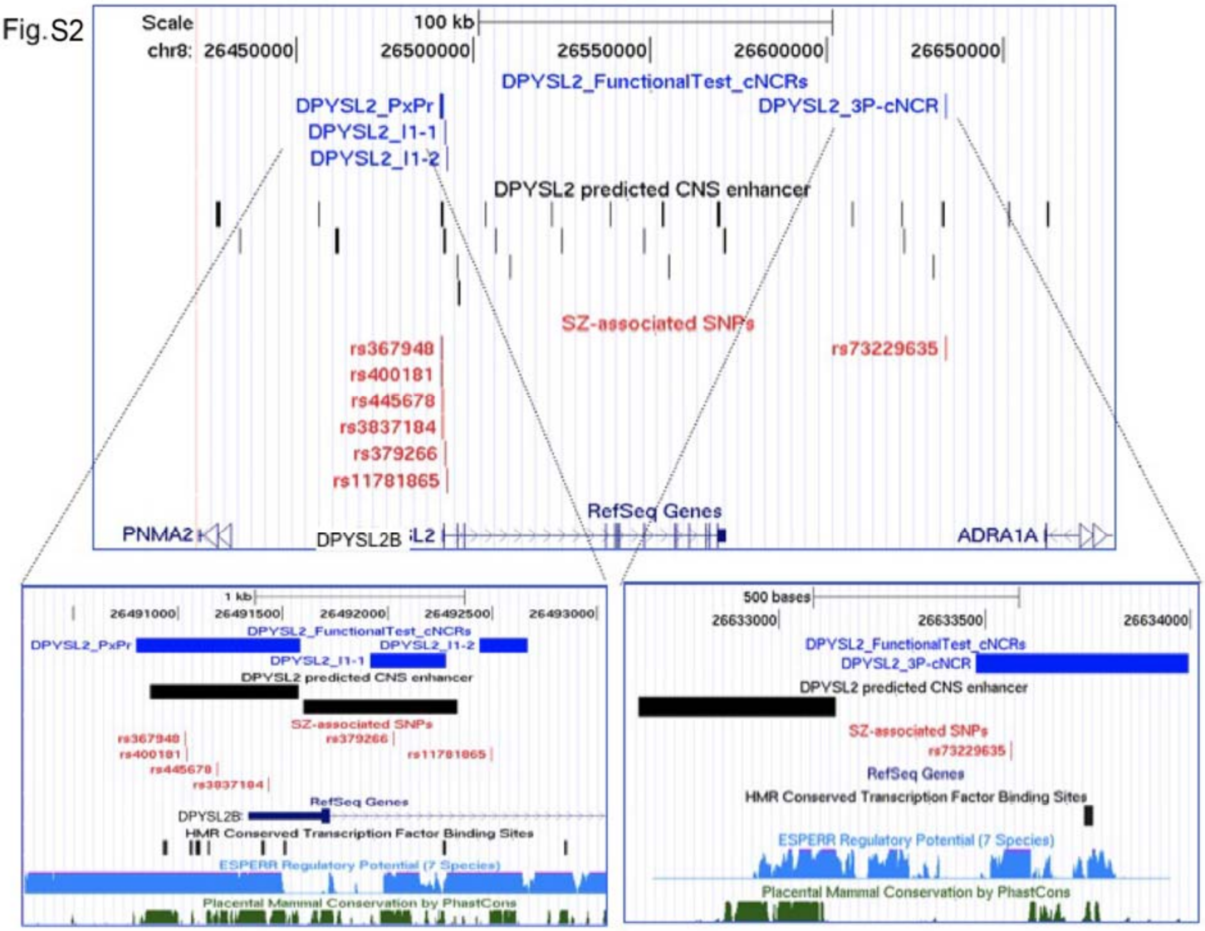

Figure S2 Four cNCRs selected for functional tests shown on UCSC genome browser in an ~260kb interval around *DPYSL2*. Blue custom track showed 4 regions (cNCRs). Black custom track showed bioinformatically predicted CNS enhancer. Red custom track showed some of those SZ-associated SNPs identified by sequencing and genotyping in the present study, and which were contained in 4 cNCRs shown in blue bars for functional tests
