# Supplementary figures and images for: Functional Variants in DPYSL2 Sequence Increase Risk of Schizophrenia and Suggest a Link to mTOR Signaling
Source: G3 (Bethesda). 2014 Nov 20;5(1):61–72. doi: 10.1534/g3.114.015636 (PMC4291470; doi:10.1534/g3.114.015636)

Fig. S3

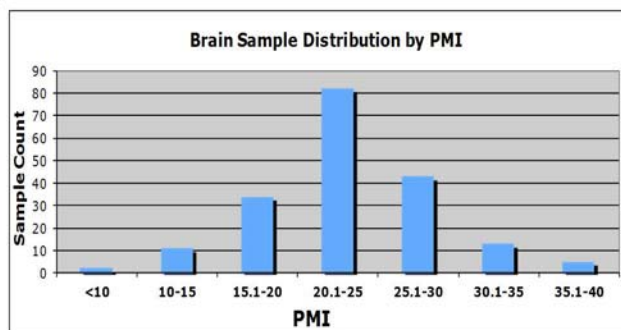

**Figure S3** Distribution of PMI from 190 brain samples

Supplement: Supporting Information [file supp_g3.114.015636_FigureS3.pdf]
