# Supplementary material for: Functional Variants in DPYSL2 Sequence Increase Risk of Schizophrenia and Suggest a Link to mTOR Signaling
Source: G3 (Bethesda). 2014 Nov 20;5(1):61–72. doi: 10.1534/g3.114.015636 (PMC4291470; doi:10.1534/g3.114.015636)
Supplement: Supporting Information [file supp_g3.114.015636_FigureS5.pdf]

Fig.S5

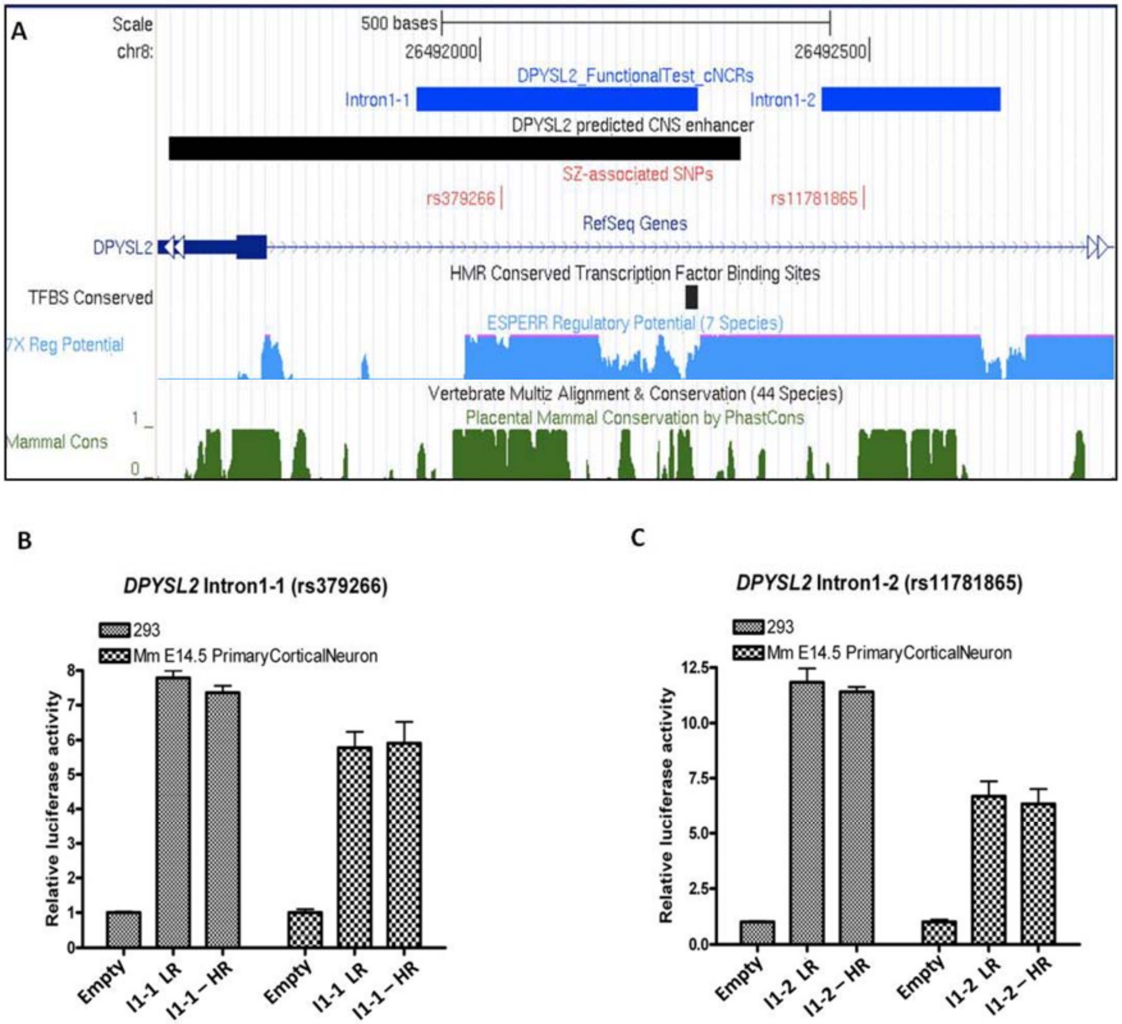

Figure S5 Human *DPYSL2* intron1 two constructs containing 2 SZ-associated SNPs did not show difference in driving luciferase expression between constructs containing Wt allele and Risk allele: A) Two constructs (blue bar) containing 2 SNPs in intron1 (red thin line) shown on the UCSC genome browser; B) *DPYSL2*_I1-1 luciferase assay results in cells; C) *DPYSL2*_I1-2 luciferase results in cells
