# Supplementary material for: Functional Variants in DPYSL2 Sequence Increase Risk of Schizophrenia and Suggest a Link to mTOR Signaling
Source: G3 (Bethesda). 2014 Nov 20;5(1):61–72. doi: 10.1534/g3.114.015636 (PMC4291470; doi:10.1534/g3.114.015636)
Supplement: Supporting Information [file supp_g3.114.015636_FigureS6.pdf]

Fig. S6

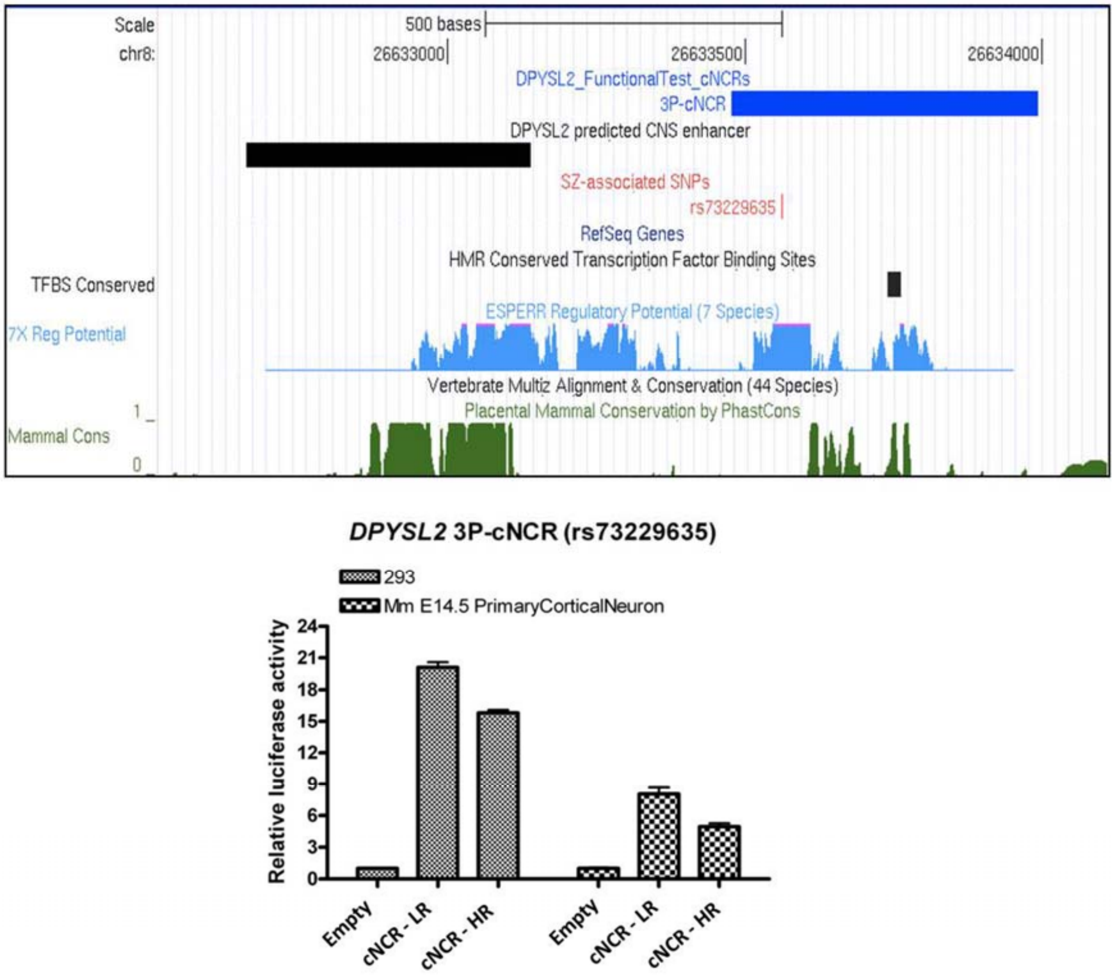

Figure S6 Human *DPYSL2* 3'-flanking cNCR (3P-cNCR) construct containing a SZ-associated SNP rs73229635 (p-value<10^{-5} in large AJ collection) showed enhancer function and difference in driving luciferase expression between constructs containing Wt allele and Risk allele: A) A construct (blue bar) containing SNP rs73229635 in 3'-flanking (red thin line) shown on the UCSC genome browser; B) 3P-cNCR luciferase assay results in cells
