# Supplementary material for: Functional Variants in DPYSL2 Sequence Increase Risk of Schizophrenia and Suggest a Link to mTOR Signaling
Source: G3 (Bethesda). 2014 Nov 20;5(1):61–72. doi: 10.1534/g3.114.015636 (PMC4291470; doi:10.1534/g3.114.015636)
Supplement: Supporting Information [file supp_g3.114.015636_FigureS7.pdf]

Fig. S7

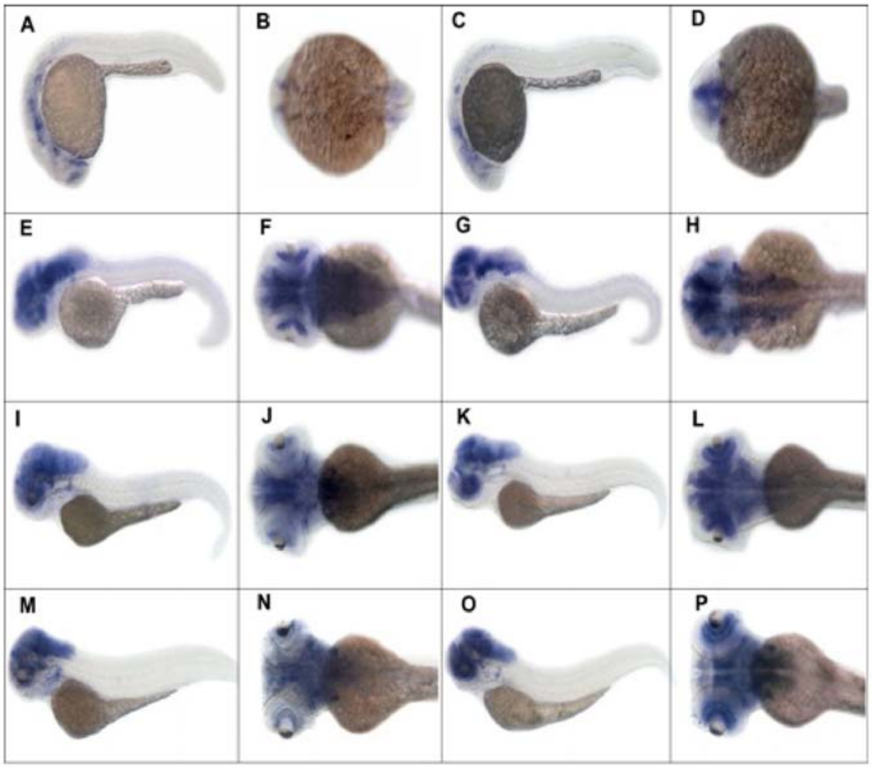

Figure S7 Whole mount *in situ* hybridization with *dpysl2a* and *dpysl2b* riboprobes. A, B, E, F, I, J, M, N – *dpysl2a in situ*; C, D, G, H, K, L, O, P – *dpysl2b in situ;* A-D – 24 hpf; E-H – 48 hpf; I-L – 72 hpf; M-P – 96 hpf; anterior is to the left; 1st and 3rd column – lateral view; 2nd and 4th column – dorsal view
