# Supplementary material for: Functional Variants in DPYSL2 Sequence Increase Risk of Schizophrenia and Suggest a Link to mTOR Signaling
Source: G3 (Bethesda). 2014 Nov 20;5(1):61–72. doi: 10.1534/g3.114.015636 (PMC4291470; doi:10.1534/g3.114.015636)
Supplement: Supporting Information [file supp_g3.114.015636_FigureS8.pdf]

Fig. S8

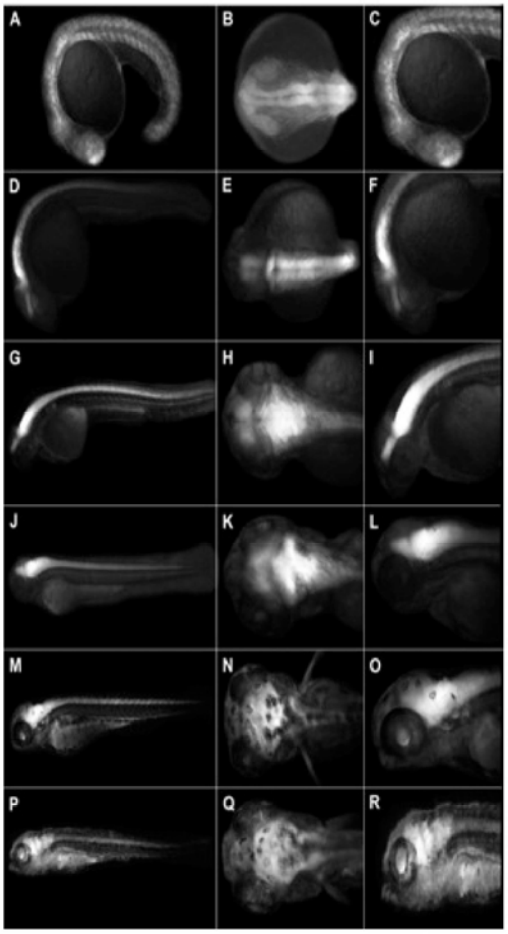

Figure S8 Transgenic zebrafish *DPYSL2*_PxPr line with *EGFP* reporter. A-C – 20 hpf; D-E – 24 hpf; G-I – 48 hpf; J-L – 72 hpf; M-O – 96hpf; P-R – 120 hpf; anterior is to the left; 1st and 3rd column – lateral view; 2nd column – dorsal view; 3rd column – lateral head magnification picture
