# Supplementary material for: Functional Variants in DPYSL2 Sequence Increase Risk of Schizophrenia and Suggest a Link to mTOR Signaling
Source: G3 (Bethesda). 2014 Nov 20;5(1):61–72. doi: 10.1534/g3.114.015636 (PMC4291470; doi:10.1534/g3.114.015636)
Supplement: Supporting Information [file supp_g3.114.015636_FigureS9.pdf]

Fig. S9

```
Mouse       ATTGCTGTAGTTTATTTCCACCCCCTTCCCTCCTGTT---------------------CT
Rat         ATTGCTGTAGTTTATTTCCACCCCCTTCCCTCCTGTT---------------------CT
Human       ATTGCTGTAGTTTATTTCCACCCCCTTCCCTCCTGTTTCTCTCTCTCCTTCTCTCTCTCT
            *************************************                     **

Mouse       CTCTCCCTCT-T-TCTTTTTTCCGCCCTAGCTGGATCTGTGTTGGAGGAGAG----GAAA
Rat         CTCTCCCTCTCTTTCTTTTTTCCGCCCTAGCTGGATCTGTGTTGGAGGAGAG----AAAA
Human       CTCTCTCTCTCTCTTTTTTTTCCGCCCTAGCTGGGGCTGTGTTGGAGGAGAGGAAGAAAG
            ***** **** * * *******************  ****************     **

Mouse       AGAGACAGAGGATTGCATCTG----------TTTTGAAATCTCCTAATAGCAAGACCAGT
Rat         AGAGACAGAGGATTGCATCTGTCTCTTATAGTTTTGAAATCTCCTAATAGCAAGACCAGC
Human       AGAGACAGAGGATTGCATTCATCCGTTACGTTCTTGAAATTTCCTAATAGCAAGACCAGC
            ******************             * ******* ******************

Mouse       TAAGGGATTGTACCTTTTTCCTAC----------AAATATAAATATATATATATTTTAAA
Rat         TAAGGGATTGTACCTTTTTCCTAC----------AAATATAAATATATATATATTTTAAA
Human       GAAGCGGTTGCACCCTTTTCAATCTTGCAAAGGAAAAAAACAAAACAAAACAAAAAAAAC
             *** * *** *** *****   *          *** *  ** * * *   *    **

Mouse       CCAAGTCTTTTTTTTTCCGGCTATCTTTGCTTTAAAGCTGTCCTCTTGAGATTACTTCCT
Rat         CCAAGTCTTTTTTT--CCGGCTCTCTTTGCTTTAAAGCTGTCCTCTTGAAATTACTTCCC
Human       CCAAGTCCCCTTCC---CGGCAGTTTTTGCCTTAAAGCTGCCCTCTTGAAATTAATTTTT
            *******   **     ****  * ***** ********* ******** **** **

Mouse       CCCGCCCCCCGGAGAGATG
Rat         CCCGCCCCCCGGAGAGATG
Human       TCC---CAGGAGAGAGATG
             **        ********
```

Figure S9 Sequences of human and rat *DPYSL2* showing 5'-TOP sequence and polymorphic DNR in human sequence
