# Supplementary material for: Functional Variants in DPYSL2 Sequence Increase Risk of Schizophrenia and Suggest a Link to mTOR Signaling
Source: G3 (Bethesda). 2014 Nov 20;5(1):61–72. doi: 10.1534/g3.114.015636 (PMC4291470; doi:10.1534/g3.114.015636)
Supplement: Supporting Information [file supp_g3.114.015636_TableS1.pdf]

Table S1 Sample counts for sequencing and genotyping

	SZ Independent cases		Independent Controls		
	CEU	AJ	CEPH	AJ	
				EpiGen	NYCP
Sequencing	48	89	96	55	
Genotyping		729		821	721
