# Supplementary material for: Functional Variants in DPYSL2 Sequence Increase Risk of Schizophrenia and Suggest a Link to mTOR Signaling
Source: G3 (Bethesda). 2014 Nov 20;5(1):61–72. doi: 10.1534/g3.114.015636 (PMC4291470; doi:10.1534/g3.114.015636)
Supplement: Supporting Information [file supp_g3.114.015636_TableS2.pdf]

Table S2   Primers for *DPYSL2* 14 exons and 27 cNCRs

Primer_ID	Primer-Seq	Genomic location	Product(bp)
DPYSL2 E1 F	AATCGCTGCTCGTCTCTCTC	chr8:26491276-26491829	554
DPYSL2 E1 R	AGTTCGGTCCCTCTCCTTTC		
DPYSL2 E2 F	GGTTGCCACATACAGGCTTA	chr8:26495239-26495602	364
DPYSL2 E2 R	TGGTCACTCTCTGATGTTCCTG		
DPYSL2 E3 F	GCACAGAGCGGAGGATAGTT	chr8:26497129-26497521	393
DPYSL2 E3 R	GTGGCTCAGGACAGCAAGTT		
DPYSL2 E4 F	CGATTTTGAACCCAAGAAGC	chr8:26537468-26537843	376
DPYSL2 E4 R	ACAAATGGGATCTGGAAGCA		
DPYSL2 E5-7-1 F1	TGCTTCCTTTACCATTCTGTG	chr8:26539859-26540409	551
DPYSL2 E5-7-1 R1	AAGCTCTTGGGTGACACCTG		
DPYSL2 E5-7-2 F	CGGCACTCAGAACCTCCTTA	chr8:26540352-26540844	493
DPYSL2 E5-7-2 R	ACCAGGTCCCTCTGCTTTTC		
DPYSL2 E5-7-3 F	CAGGGCTTTCTTTTTCGTCA	chr8:26540732-26541264	533
DPYSL2 E5-7-3 R	ATTTTGCACAGCTCTGATGG		
DPYSL2 E5-7-4 F	GAACGATCGGCAGTGGTAAT	chr8:26541186-26541597	412
DPYSL2 E5-7-4 R	ATCTTCTGGCGAAACAGTCC		
DPYSL2 E8 F	CAGCCTCGCCTTCATCTTAG	chr8:26548111-26548535	425
DPYSL2 E8 R	ATACTAACAAGGCCGCAGCA		
DPYSL2 E9 F	TCTGCGAGATGAGCCTGATA	chr8:26556732-26557152	421
DPYSL2 E9 R	TTTGGTGAACAAGGGTCTCC		
DPYSL2 E10 F	GGGAGGGGATTCTGGATAAA	chr8:26557201-26557618	418
DPYSL2 E10 R	TGTCATTTCAAGGCCTCCAT		
DPYSL2 E11 F	CAACGCTCTTGACATCCATC	chr8:26560945-26561380	436
DPYSL2 E11 R	CCCAGCAAATTGCAAATCAT		
DPYSL2 E12 F	CAGGATCCCTGTCTCTGAGTCT	chr8:26565570-26565997	428
DPYSL2 E12 R	GCTCCCTCTTATCCCTGGAA		
DPYSL2 E13 F	CACACAACACCTGTCCACCT	chr8:26566525-26566956	432
DPYSL2 E13 R	CACGTTGGAGAGAAAGGGAAT		
DPYSL2 E14 F	GCACTTTTCCTCCTGAGCTG	chr8:26568950-26569384	435
DPYSL2 E14 R	GGTGGGTAGTCTTGGGTGTG		

cNCR1_-70.5_F	CTGCAGTGAGCCGAGATTTT	chr8:26,420,806-26,423,180	2375
cNCR1_-70.5_R	TGCATTAACGGTCTTCCTACTG		
cNCR2_-64.4_F	AAGGCGAGTGGACAAACTGA	chr8:26,426,916-26,428,447	1532
cNCR2_-64.4_R	CGGGAGGGTTTCAGAAGAAT		
cNCR3_-30.5_F	TACTGGAGCAGAAGCCCACT	chr8:26,460,813-26,462,243	1431
cNCR3_-30.5_R	GACCACCAGGCTCAAGTGAT		
cNCR4_-1.2_F	CTGGGCAGTCAGGACACTCT	chr8:26,490,109-26,492,741	2633
cNCR4_-1.2_R	GAGAGGTGGGAGCAAGACTG		
cNCR5_+11.9_F	AAATCACATGGTCCCAGCAC	chr8:26,503,272-26,503,801	530
cNCR5_+11.9_R	CCCGTGTCCTTAGGAGAAATG		
cNCR6_+15.0_F	CCCCAAGCTGACCTTTCTCT	chr8:26,506,341-26,507,564	1223
cNCR6_+15.0_R	CACGCTCAGACAGAAAGCAC		
cNCR7_+17.6_F	GCATTCAAATGAGGCCAACT	chr8:26,508,894-26,511,818	2925
cNCR7_+17.6_R	TCAGGAGATGGAGAGGAGGA		
cNCR8_+30.5_F	GAGGGATACTTTGCATCTGGA	chr8:26,521,874-26,52,3744	1871
cNCR8_+30.5_R	TCCCAACAGAATGTATCAGGAA		
cNCR9_+54.8_F	CTGTATCTCTGGGAGGTGCTG	chr8:26,546,129-26,546,478	350
cNCR9_+54.8_R	CCTTGTCTCCACCAGAGCTT		
cNCR10_+62.1_F	CTGTCCATCCCAGTTGGTTT	chr8:26,553,478-26,554,076	599
cNCR10_+62.1_R	CAGGCCTCTACCTCCACAAG		
cNCR11_+63.7_F	GATGAACCGGAGTGGTTTGT	chr8:26,555,009-26,555,563	555
cNCR11_+63.7_R	TCCAAGCTGGAATTTGATCC		
cNCR12_+71.1_F	GGGTAGGTTGGGGTGAAAAT	chr8:26,562,422-26,562,930	509
cNCR12_+71.1_R	TGACTTCTTGGGTGTCCTGA		
cNCR13_+77.9_F	TACTGTGGAGCAGCCAGTTC	chr8:26,569,258-26,570,280	1023
cNCR13_+77.9_R	GGTTCTGCAGCCTCATACCT		
cNCR14_+79.4_F	TCCATCCTTCCCTCTCCTTT	chr8:26,570,707-26,571,758	1052
cNCR14_+79.4_R	TAGCAGCCTGTGACATGGAG		
cNCR15_+80.3_F	TGTGGTTGTTTCAGGGGTGT	chr8:26,571,596-26,572,220	625
cNCR15_+80.3_R	CCCAAGGCACACACTCTCTT		
cNCR16_+81.1_F	ATATTGCCTGGGCTGGTTTT	chr8:26,572,480-26,573,107	628
cNCR16_+81.1_R	CCCATCTTGGTGTGAGATGA		

cNCR17_+83.2_F	AGCCAAGATCGTGCCACTTA	chr8:26,574,557-26,575,013	457
cNCR17_+83.2_R	CCTGTGTGGTGCAGAGATGT		
cNCR18_+85.0_F	TGGTAATGGCCGCTTAAGTT	chr8:26,576,327-26,576,849	523
cNCR18_+85.0_R	TACAGGCTGTCCCTGTCCTT		
cNCR19_+100.5_F	GAGGGTCTCCTTCCTGCTCT	chr8:26,591,864-26,592,384	521
cNCR19_+100.5_R	CTGGAATTGGGAGGGAAGTT		
cNCR20_+114.8_F	AGATCAGGCCTCCTCTACCC	chr8:26,606,089-26,607,484	1396
cNCR20_+114.8_R	CAGCGATATGCAAAATGAGG		
cNCR21_+122.3_F	CTCCCGAGTTGCTTGTGAAT	chr8:26,613,669-26,614,069	401
cNCR21_+122.3_R	TCAGGACTTTCAAGGCTGCT		
cNCR22_+128.3_F	CTGCACCCAGTGATGTGTGT	chr8:26,619,609-26,621,844	2236
cNCR22_+128.3_R	TGCTGTTGTCCTCTGTGAAAA		
cNCR23_+138.3_F	AAGCATTCTCTGTGGGAGTCA	chr8:26,630,003-26,630,401	399
cNCR23_+138.3_R	TTCCCATCTTTTGCAGTAGGA		
cNCR24_+141.0_F	GAGTTTGCCTCTGGGAAGTG	chr8:26,632,377-26,633,994	1618
cNCR24_+141.0_R	CTGGCTTTTGATCTCGGTTC		
cNCR25_+149.6_F	CCTTCCTGCCAATTCTCTGA	chr8:26,640,955-26,641,598	644
cNCR25_+149.6_R	TCCCAAAGTGCTGGGATTAG		
cNCR26_+151.0_F	CCCCCACACCTTTCTTTCTT	chr8:26,642,317-26,642,906	590
cNCR26_+151.0_R	TACTAATACAAGCCTTATACCCTGTGC		
cNCR27_+156.3_F	GCACCCTATTCTCTCTCCTCCT	chr8:26,647,655-26,649,235	1581
cNCR27_+156.3_R	GGCCTTTGAAGGAAACCTCT		
