# Supplementary material for: Functional Variants in DPYSL2 Sequence Increase Risk of Schizophrenia and Suggest a Link to mTOR Signaling
Source: G3 (Bethesda). 2014 Nov 20;5(1):61–72. doi: 10.1534/g3.114.015636 (PMC4291470; doi:10.1534/g3.114.015636)
Supplement: Supporting Information [file supp_g3.114.015636_TableS3.pdf]

Table S3 Four cNCRs selected for functional tests

cNCR Name<br>Genomic coordinates	Contruct name	SNPs covered	Allele(s)of SNPs	Length (bp)
***DPYSL2*_PxPr**<br>26490803-26491584	DPYSL2_Pr3SNP_LR<br>DPYSL2_Pr3SNP_HR	rs367948, rs400181<br>rs445678, rs3837184	CTT+11TC<br>GCC+11TC	782
	DPYSL2_5'DNR_LR	rs367948, rs400181<br>rs445678, rs3837184	CTT+11TC<br>GCC+11TC	782
	DPYSL2_5'DNR_HR	rs367948, rs400181<br>rs445678, rs3837184	CTT+12TC<br>GCC+12TC<br>CTT+13TC<br>GCC+13TC<br>CTT+14TC<br>GCC+14TC	
***DPYSL2*_I1-1**<br>26491918-26492279	DPYSL2_I1-1_LR<br>DPYSL2_I1-1_HR	rs379266	T<br>C	362
***DPYSL2*_I1-2**<br>26492439-26492669	DPYSL2_I1-2_LR<br>DPYSL2_I1-2_HR	rs11781865	G<br>C	231
***DPYSL2*_3P-cNCR**<br>26633478-26633994	DPYSL2_3P-cNCR_LR<br>DPYSL2_3P-cNCR_HR	rs73229635	T<br>C	517

note: LR stands for low risk; HR stands for high risk
