# Supplementary material for: Functional Variants in DPYSL2 Sequence Increase Risk of Schizophrenia and Suggest a Link to mTOR Signaling
Source: G3 (Bethesda). 2014 Nov 20;5(1):61–72. doi: 10.1534/g3.114.015636 (PMC4291470; doi:10.1534/g3.114.015636)
Supplement: Supporting Information [file supp_g3.114.015636_TableS4.pdf]

Table S4 Brain samples

Distribution by sex and plates

SEX	Plate1	Plate2	Total
Female	22	21	43
Male	75	72	147
Total	97	93	190

Distribution by age and plates

AGE	Plate1	Plate2	Total
<20	2	0	2
21-30	2	2	4
31-40	1	2	3
41-50	5	5	10
51-60	31	28	59
61-70	28	29	57
71-80	24	24	48
>80	4	3	7
Total	97	93	190
