# Supplementary material for: Functional Variants in DPYSL2 Sequence Increase Risk of Schizophrenia and Suggest a Link to mTOR Signaling
Source: G3 (Bethesda). 2014 Nov 20;5(1):61–72. doi: 10.1534/g3.114.015636 (PMC4291470; doi:10.1534/g3.114.015636)
Supplement: Supporting Information [file supp_g3.114.015636_TableS5.pdf]

Table S5 Primers used for real time PCR

Primer Name	Primer sequence	Length(bp)	Genome coordinates (hg18)
Human_DPYSL2B_F	AAAATATTCCACGCATCACG	113bp (DPYSL2B_F/R)	chr8:26491707-26491726
Human_DPYSL2A_F	AAGCCCTGCAGAACATCAAC	119bp (DPYSL2A_F/R)	chr8:26428087-26428106
Human_DPYSL2_R	TTGCTTGATCAACCCATCTTC		chr8:26495474-26495494
