# Supplementary material for: Functional Variants in DPYSL2 Sequence Increase Risk of Schizophrenia and Suggest a Link to mTOR Signaling
Source: G3 (Bethesda). 2014 Nov 20;5(1):61–72. doi: 10.1534/g3.114.015636 (PMC4291470; doi:10.1534/g3.114.015636)
Supplement: Supporting Information [file supp_g3.114.015636_TableS6.pdf]

Table S6 The list of 120 variants in and around *DPYSL2* identified by sequencing

Variant	Variant ID	Sequence context	genomic coordinate (dbSNP129)	genomic coordinate (dbSNP135)
1	rs2233699	5' end cNCR	26422670	26366753
2	rs17403251	5' end cNCR	26427460	26371543
3	rs149517015	5' end cNCR	26428268	26372351
4	rs9314324	5' end cNCR	26460877	26404960
5	rs13279683	5' end cNCR	26460941	26405024
6	rs78025765	5' end cNCR	26461102	26405185
7	rs9314325	5' end cNCR	26461170	26405253
8	rs7831864	5' end cNCR	26461425	26405508
9	rs12548421	5' end cNCR	26461547	26405630
10	rs12543392	5' end cNCR	26461556	26405639
11	rs12543398	5' end cNCR	26461608	26405691
12	rs10110479	5' end cNCR	26461939	26406022
13	rs431246	5' end cNCR(PxPr)	26490712	26434795
14	rs367948	5' end cNCR(PxPr)	26491033	26435116
15	rs400181	5' end cNCR(PxPr)	26491040	26435123
16	rs445678	5' end cNCR(PxPr)	26491188	26435271
17	rs3837184	5'-UTR DNR	26491428	26435511
18	rs58966940	5'-UTR	26491458	26435541
19	rs379266	intronic cNCR	26492026	26436109
20	rs11285674	intronic cNCR	26492389	26436472
21	rs11781865	intronic cNCR	26492493	26436576
22	rs2584184	intronic cNCR	26492687	26436770
23	rs415524	intronic cNCR	26497192	26441275
24	rs11786691	coding synonymous	26497394	26441477
25	rs408753	intronic cNCR	26497479	26441562
26	rs17055482	intronic cNCR	26503398	26447481
27	rs140053560	intronic cNCR	26503656	26447739
28	rs62491913	intronic cNCR	26503707	26447790
29	rs68019818	intronic cNCR	26503747	26447830
30	rs7825468	intronic cNCR	26506390	26450473
31	rs146056239	intronic cNCR	26506775	26450858
32	rs5029306	intronic cNCR	26509022	26453105
33	rs7820433	intronic cNCR	26521976	26466059
34	rs113199330	coding synonymous	26537583	26481666
35	rs327222	coding synonymous	26537688	26481771
36	rs139123217	coding synonymous	26537739	26481822
37	rs2289592	intronic cNCR	26540291	26484374
38	un26540309	intronic cNCR	26540309	26484392
39	un26540321	intronic cNCR	26540321	26484404
40	rs17322275	intronic cNCR	26540530	26484613
41	rs327228	intronic cNCR	26540979	26485062
42	rs73678824	intronic cNCR	26540980	26485063
43	R227R	coding synonymous	26541364	26485447
44	rs753508	intronic cNCR	26546168	26490251

45	rs327217	intronic cNCR	26546233	26490316
46	rs35621323	coding synonymous	26548248	26492331
47	rs78121726	coding synonymous	26548314	26492397
48	rs148064770	coding nonsynonymous	26548321	26492404
49	rs55906521	intronic cNCR	26548387	26492470
50	rs1867042	intronic cNCR	26548477	26492560
51	rs77434938	intronic cNCR	26553737	26497820
52	rs13277175	intronic cNCR	26553930	26498013
53	rs327218	intronic cNCR	26555144	26499227
54	rs118022714	intronic cNCR	26555248	26499331
55	rs62493399	intronic cNCR	26555414	26499497
56	rs139163162	coding synonymous	26561224	26505307
57	rs11776801	intronic cNCR	26561368	26505451
58	rs9644117	intronic cNCR	26561435	26505518
59	rs708621	coding synonymous	26566709	26510792
60	rs147699216	coding synonymous	26569064	26513147
61	un26569149	3'-UTR cNCR	26569149	26513232
62	rs58827210	3'-UTR cNCR	26570018	26514101
63	rs17055639	3'-UTR cNCR	26570061	26514144
64	rs17055641	3'-UTR cNCR	26570088	26514171
65	rs1058332	3'-UTR cNCR	26570210	26514293
66	rs11863	3'-UTR cNCR	26570233	26514316
67	rs45471201	3'-UTR cNCR	26571055	26515138
68	rs17666	3'-UTR cNCR	26571375	26515458
69	rs10495	3'-UTR cNCR	26571389	26515472
70	rs10042	3'-UTR cNCR	26571580	26515663
71	rs7827731	3'-end cNCR	26571810	26515893
72	rs7828056	3'-end cNCR	26571955	26516038
73	rs7831883	3'-end cNCR	26572130	26516213
74	rs57045236	3'-end cNCR	26572888	26516971
75	rs7845740	3'-end cNCR	26574668	26518751
76	rs7842128	3'-end cNCR	26574697	26518780
77	rs58047393	3'-end cNCR	26574771	26518854
78	rs57846118	3'-end cNCR	26574826	26518909
79	rs56322416	3'-end cNCR	26574827	26518910
80	rs12155555	3'-end cNCR	26576477	26520560
81	rs6557935	3'-end cNCR	26576551	26520634
82	rs186111033	3'-end cNCR	26592043	26536126
83	un26592058	3'-end cNCR	26592058	26536141
84	rs7831201	3'-end cNCR	26592060	26536143
85	rs187288868	3'-end cNCR	26606195	26550278
86	rs112917653	3'-end cNCR	26606207	26550290
87	rs73678614	3'-end cNCR	26606441	26550524
88	un26606675	3'-end cNCR	26606675	26550758
89	un26606723	3'-end cNCR	26606723	26550806
90	rs17330796	3'-end cNCR	26607314	26551397
91	rs17055703	3'-end cNCR	26607333	26551416
92	un26613860	3'-end cNCR	26613860	26557943
93	rs17422356	3'-end cNCR	26614030	26558113

94	rs115507492	3'-end cNCR	26621427	26565510
95	rs191918596	3'-end cNCR	26621583	26565666
96	rs7007717	3'-end cNCR	26621603	26565686
97	rs7001655	3'-end cNCR	26621687	26565770
98	rs34790461	3'-end cNCR	26621771	26565854
99	rs13278281	3'-end cNCR	26632938	26577021
100	rs73229635	3'-end cNCR	26633562	26577645
101	rs114104363	3'-end cNCR	26633800	26577883
102	rs994098	3'-end cNCR	26633836	26577919
103	rs1036813	3'-end cNCR	26633934	26578017
104	rs6998714	3'-end cNCR	26641066	26585149
105	rs4732831	3'-end cNCR	26641094	26585177
106	un26641095	3'-end cNCR	26641095	26585178
107	rs10503796	3'-end cNCR	26641185	26585268
108	rs1908650	3'-end cNCR	26641530	26585613
109	rs1025859	3'-end cNCR	26647800	26591883
110	rs78167668	3'-end cNCR	26648009	26592092
111	rs1025858	3'-end cNCR	26648036	26592119
112	rs2132449	3'-end cNCR	26648270	26592353
113	rs1016733	3'-end cNCR	26648327	26592410
114	rs1016734	3'-end cNCR	26648421	26592504
115	rs76802184	3'-end cNCR	26648455	26592538
116	rs35551735	3'-end cNCR	26648560	26592643
117	rs17055838	3'-end cNCR	26649076	26593159
118	rs111576315	3'-end cNCR	26649096	26593179
119	rs191064950	3'-end cNCR	26649117	26593200
120	un26649188	3'-end cNCR	26649188	26593271

Note: novel variants are named after their genomic coordinates in dbSNP129
